# Best billiard ball in the 19th century: Composite materials made of celluloid and bone as substitutes for ivory

**DOI:** 10.1093/pnasnexus/pgad360

**Published:** 2023-11-03

**Authors:** Artur Neves, Robert Friedel, Maria J Melo, Maria Elvira Callapez, Edward P Vicenzi, Thomas Lam

**Affiliations:** REQUIMTE—Laboratório Associado para a Química Verde and Department of Conservation and Restoration, Faculdade de Ciências e Tecnologia, Universidade NOVA de Lisboa, Campus Caparica, Monte de Caparica 2829-516, Portugal; Department of History, University of Maryland, 2115 Francis Scott Key Hall, College Park, Maryland 20742, USA; REQUIMTE—Laboratório Associado para a Química Verde and Department of Conservation and Restoration, Faculdade de Ciências e Tecnologia, Universidade NOVA de Lisboa, Campus Caparica, Monte de Caparica 2829-516, Portugal; Centro Interuniversitário de História das Ciências e da Tecnologia, Faculdade de Ciências, Universidade de Lisboa, Campo Grande, Lisbon 1749-016, Portugal; Museum Conservation Institute, Smithsonian Institution, 4120 Silver Hill Road, Suitland, Maryland 20746, USA; Museum Conservation Institute, Smithsonian Institution, 4120 Silver Hill Road, Suitland, Maryland 20746, USA

**Keywords:** ivory substitution, polymer composites, celluloid, material culture, cultural heritage, Physical Sciences, Chemistry

## Abstract

The demystification of how 19th-century novelly designed materials became significant elements of modern technological, economic, and cultural life requires a complete understanding of the material dimensions of historical artifacts. The objects frequently described as the earliest manufactured plastic products—the billiard balls made by John Wesley Hyatt and his associates from the late 1860s—are examined closely for the first time and are found to be more complex and functionally more successful than has been described. Modern analytical techniques such as optical microscopy, scanning electron microscope—energy dispersive X-ray spectroscopy, X-ray fluorescence, micro-Fourier transformed infrared, and handheld/micro-Raman spectroscopies were used to reveal the complex composition of the Smithsonian Institution's “original” 1868 celluloid billiard ball. Comparisons with billiard and pool balls commercialized from the 1880s to the 1960s showed an unexpected consistency in material formulations. All specimens were made of an unprecedented composite material prepared with a mixture of cellulose nitrate, camphor, and ground bone; the source of the bone was identified as cattle by peptide mass fingerprint (ZooMS). Patent specifications and contemporary journal descriptions explained how and when these formulations emerged. Combining the technical analyses of compositions with a careful reading of the historical record and contemporary descriptions reveals the key elements of the first successful efforts to substitute materials to assist the survival of endangered animals.

**Significance Statement**

Previous discussions of the earliest efforts to replace scarce or endangered natural materials with artificial materials have overlooked the complexity and success of these efforts. The 155-y-old billiard ball invented by John Wesley Hyatt turns out to be a pioneering example of one of the most significant types of materials in the 20th century and beyond—reinforced polymer composites. A multianalytical approach incorporating spectroscopic techniques and peptide mass fingerprint revealed the development and persistence of a composite made of ground bone, cellulose nitrate, and camphor, hereby called “reinforced celluloid”. The success of this material in providing a useful and popular alternative to elephant ivory has not been sufficiently appreciated.

## Introduction

Previous discussions of the origins and early development of plastics have overlooked the importance of carefully formulated composite materials. A more careful, analytically informed investigation of early plastic artifacts reveals more complex and interesting materials, important precedents for the emergence of designed and engineered materials in the 20th century. An appreciation of the complexity of these materials and the care with which they were designed for specific purposes has important implications for both their historical interpretation and the challenges they pose for conservation. Billiard balls have long been understood to have inspired the first development of important commercial plastics, but their carefully designed composition has not been understood. The first systematic effort to analyze this composition and to understand their development over time corrects long-held misconceptions about the early plastics and their entry into commerce and culture.

Billiards depended fundamentally on the mechanical behavior of the colliding balls, which by the 19th century were almost invariably made from elephant ivory. Because of the growing demand, ivory billiard ball suppliers were under considerable pressure, leading to calls for ivory replacements in the 1860s. In 1864, Phelan & Collender, one of the largest manufacturers of billiard suppliers at that time, published a $10,000 reward challenge for an ivory billiard ball substitute with the following requirements: “the material or composition of the artificial ivory must possess the qualities of *elasticity*, *density* and *hardness*, (…) easily turned of a perfect spherical form in the lathe, (…) readily colored and polished. It must not shrink, warp, or crack under ordinary variations of atmospheric temperature. Its specific gravity must be equal to that of natural ivory, (…) Its cost (…) must be at least *fifty per cent less*” (1). This call spurred the efforts of a young Albany, New York printer, John Wesley Hyatt (1837–1920) (2–4).

**Fig. 1.**
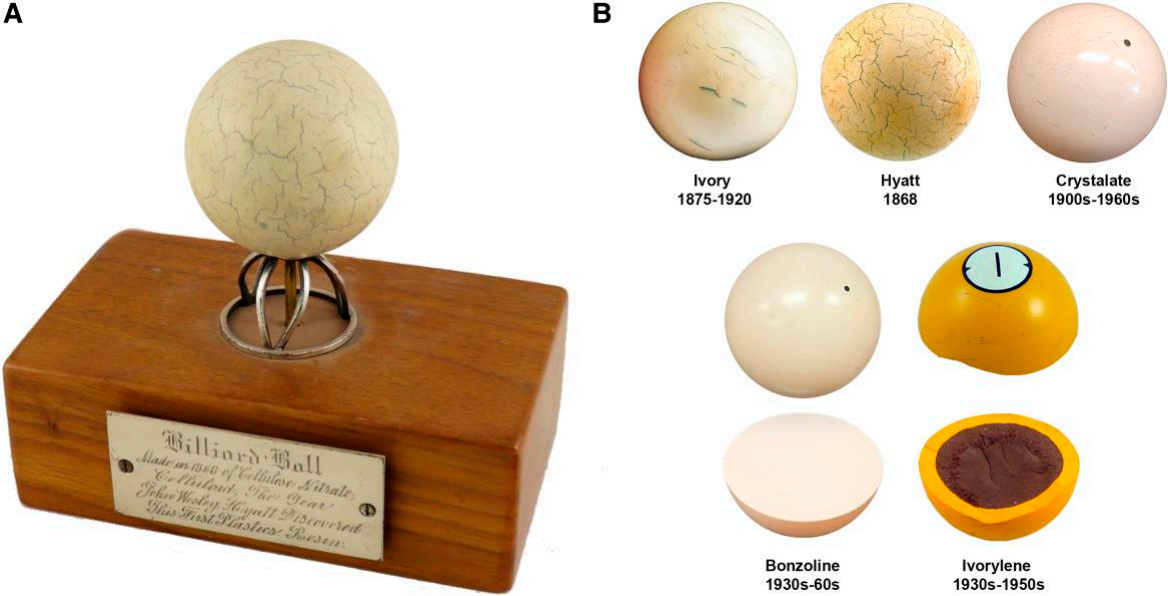
Historical billiard balls studied. A) John Wesley Hyatt and 1868 “original” celluloid billiard ball. Image credit: National Museum of American History, Smithsonian Institution (ID Number CH.334572). B) Billiard balls studied: ivory, Hyatt 1868, crystalate, bonzoline, and ivorylene. The ivory billiard ball is from the Smithsonian Collection (ID number CL.329507). Bonzoline and crystalate resemblance with ivory is evident. The interior of the bonzoline and ivorylene are shown.

In the collections of the National Museum of American History (NMAH) in Washington, DC, one can find a single billiard ball, with its own wooden pedestal, and a plaque reading: “Made in 1868 of Cellulose Nitrate, Celluloid, The Year John Wesley Hyatt Discovered This First Plastics Resin” (Fig. 1A). This billiard ball looms large in the mythology of synthetic chemistry and materials innovation. However, as billiard balls, celluloid has been dismissed as an impractical failure. Because of apocryphal stories, it is even common to think that celluloid billiard balls exploded upon contact, a myth promoted on occasion by Hyatt himself (5). Billiard balls have been seen only as a misstep in Hyatt's development of the first practical artificial plastic, celluloid, a mixture of cellulose nitrate (CN) and camphor, which succeeded in just about every other application (2, 3, 6–10).

The history of the Hyatt's billiard balls is much more interesting, but revealing their larger significance for the history of science and technology required attention to what these billiard balls were made of. To understand the materials and methods used in the manufacture of Hyatt's 1868 billiard ball, we used a multianalytical approach consisting of in situ handheld Raman and X-ray fluorescence (XRF) spectroscopy, completed with an analysis of microsamples utilizing a set of complementary methods, including micro-Fourier transformed infrared spectroscopy (μFTIR), micro-Raman spectroscopy (μRaman), scanning electron microscopy with energy dispersive X-ray spectrometry (SEM-EDS), and Zooarcheology by mass spectrometry (ZooMs, peptide mass fingerprint).

To investigate the success of Hyatt's 1868 innovation as a replacement for ivory, a comparison with other billiard balls of the late 19th and early 20th centuries was necessary. Popular guides on the practice of billiards frequently referenced the use of “composition” billiard balls, such as “bonzoline” and “crystalate,” which were acquired, and their composition determined (Fig. 1B) (11–16). Bonzoline was introduced by J.W. Hyatt's Albany Billiard Ball Company, New York, in the 1880s. Crystalate was invented by George Burt, a former Albany Billiard Ball Co. worker, who established the Crystalate Manufacturing Company in Kent, England, to produce this material in 1900 (17).

An analysis of NMAH's yellow pool ball sold under the name “ivorylene” from the 1930s to the 1950s, by Brunswick-Balke-Collender (B-B-C), the former Phelan & Collender, allowed a broader understanding of Hyatt's invention impacts in cue sports (Fig. 1B). This ball presented a yellow shell and a brown core. In 1882, Hyatt devised a method to create a billiard ball with a core-shell construction with interior and exterior segments made from different materials (US Patent [USP] 259984; Fig. S1). According to the patent description, a core or interior part of “inexpensive material” lowered the overall costs. While the traditional game, carom billiards, was played with 3 balls, pool was played with 16 balls (snooker with 22) and, therefore, the economic implications were significant.

Hyatt's first response to the Phelan & Collender 1864 challenge was a process patented in 1865 (USP 50359) that described a combination of pressed shellac with bone or ivory dust (Table S1). Then, on 1869 April 6 (USP 88633 and 88634), he patented two different processes: (i) related to the invention of a composite material involving the use of a filler, such as paper and leather chips, and an adhesive gum, where shellac is mentioned, and (ii) the dipping of a composition ball (probably made with the shellac composite) in a solution of collodion, i.e. a CN solution in diethyl ether and acetone. Finally, on 1869 May 4 (USP 89582), Hyatt patented a process for manufacturing imitation ivory by mixing ivory or bone dust with CN in a proportion of 75/25% by weight. The experimental data obtained in this study evidence this last patent as the pioneer method for developing an unprecedented bone–celluloid (CN and camphor) composite, which would successfully compete with elephant ivory billiard balls.

## Results

### Hyatt's 1868 billiard ball

**Fig. 2.**
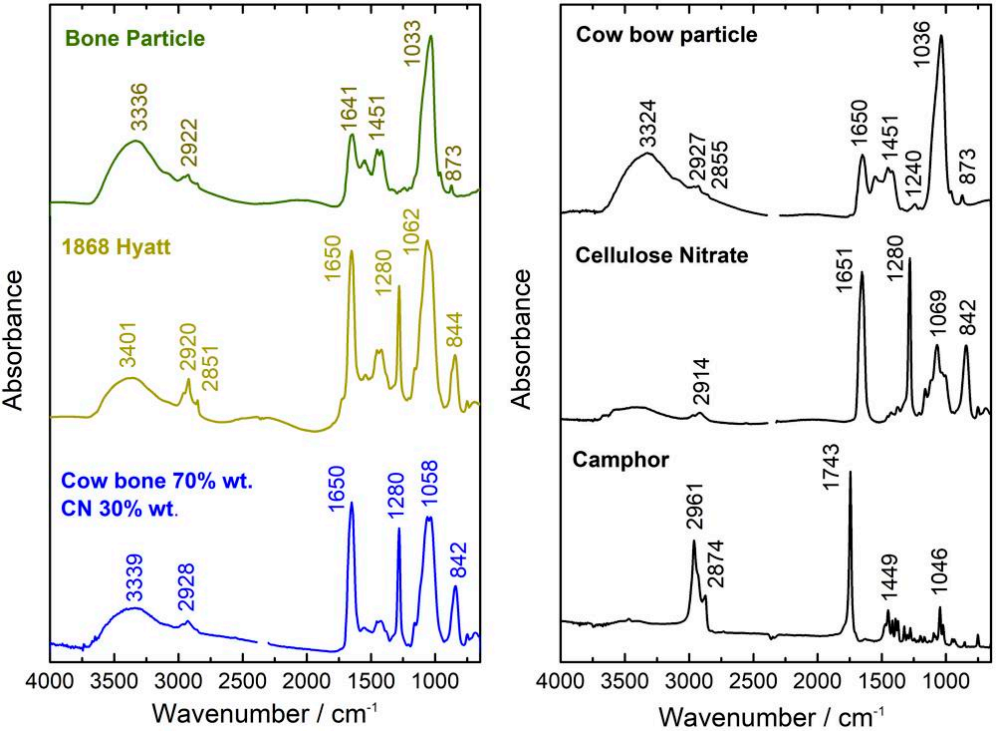
Molecular characterization of the 1868 Hyatt billiard. Left: infrared spectra of a bone particle from the 1868 billiard ball surface (top), of the average of three microsamples acquired from the surface (middle), compared with a reference prepared with 70% ground cow bone and 30% pure CN by weight (bottom). Right: infrared spectra of the references used for the development of the calibration curves for formulation quantification.

**Fig. 3.**
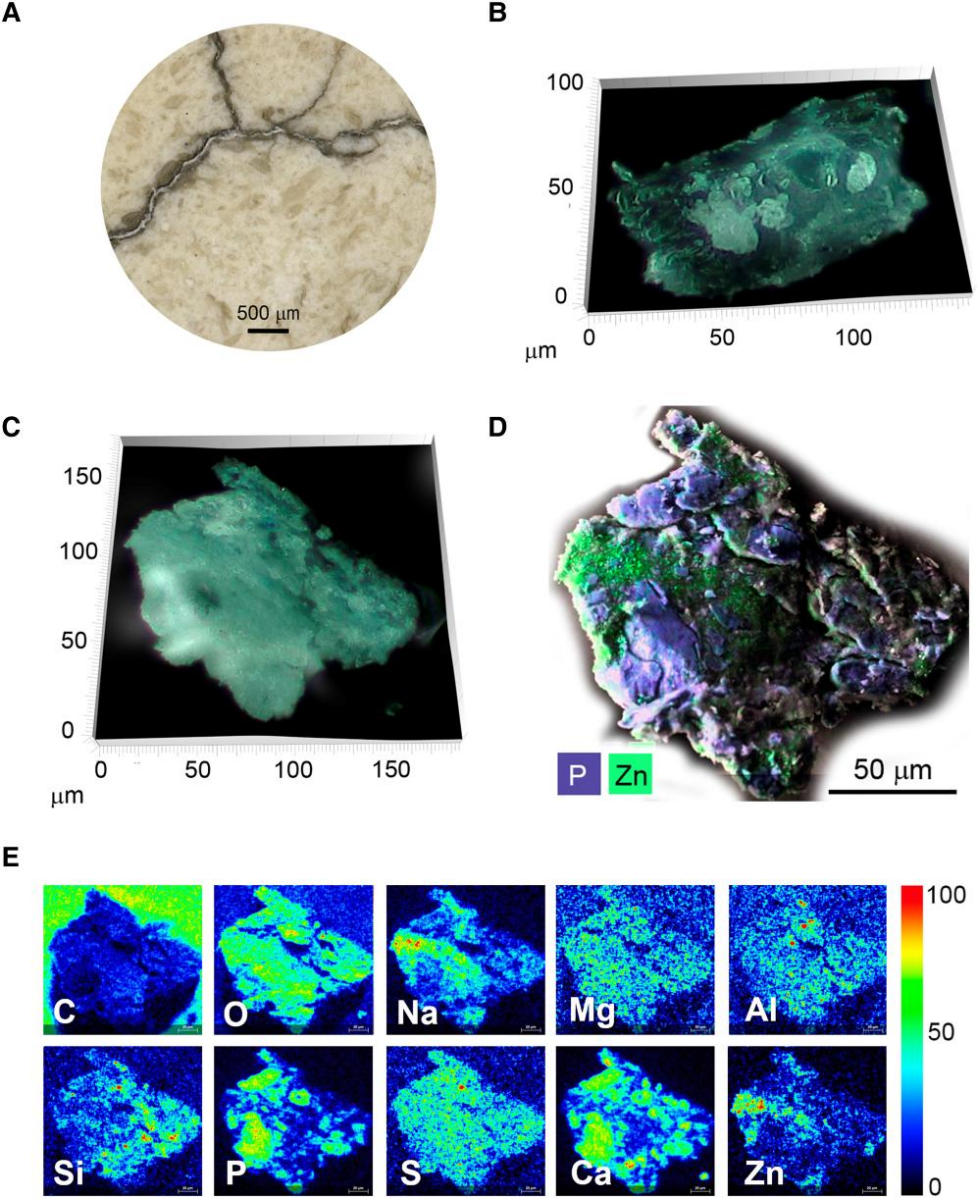
Microscopy of the 1868 Hyatt billiard ball. A) Microscope image of the surface showing its heterogenous granular surface and cracking (Hyrox Digital Microscope). B) Bone particle by 3D light microscopy. C) Bone–celluloid composite sample by 3D light microscopy. D) SEM-EDS false-color composite image showing the distribution of phosphorous (represented as blue) and zinc (represented as green). E) Compositional distribution of major components as single-element images, where the X-ray intensity is represented in rainbow scale, including carbon (C), oxygen (O), sodium (Na), magnesium (Mg), aluminum (Al), silicon (Si), phosphorous (P), sulfur (S), calcium (Ca), and zinc (Zn), by SEM-EDS.

**Fig. 4.**
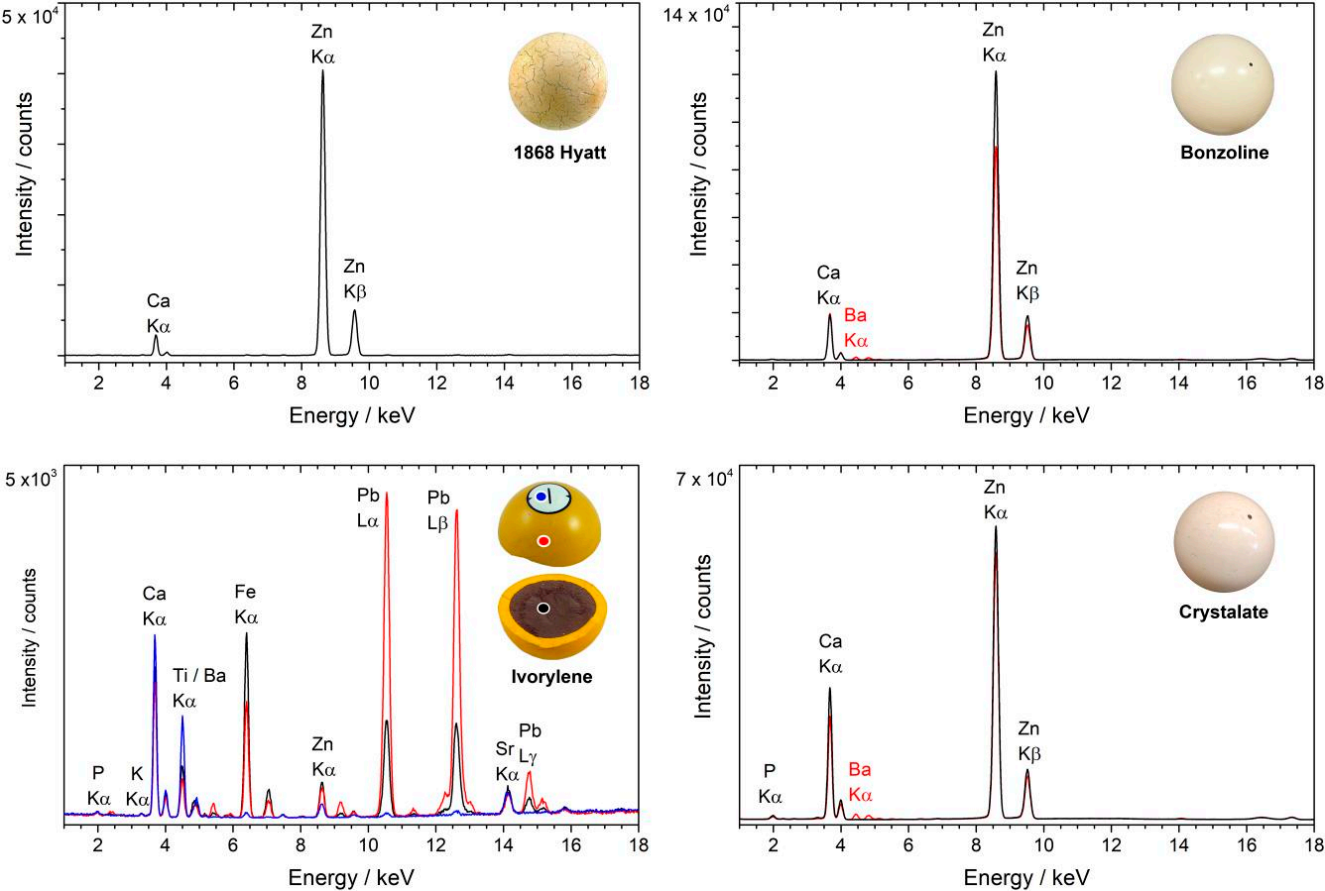
XRF spectra for 1868 Hyatt, bonzoline, and crystalate billiard balls and ivorylene pool ball. The bonzoline and crystalate spectra were collected on white and red balls. On the ivorylene, three different areas were studied: the white region where the ball number is found (blue), the yellow layer (red), and the brown core (black).

**Fig. 5.**
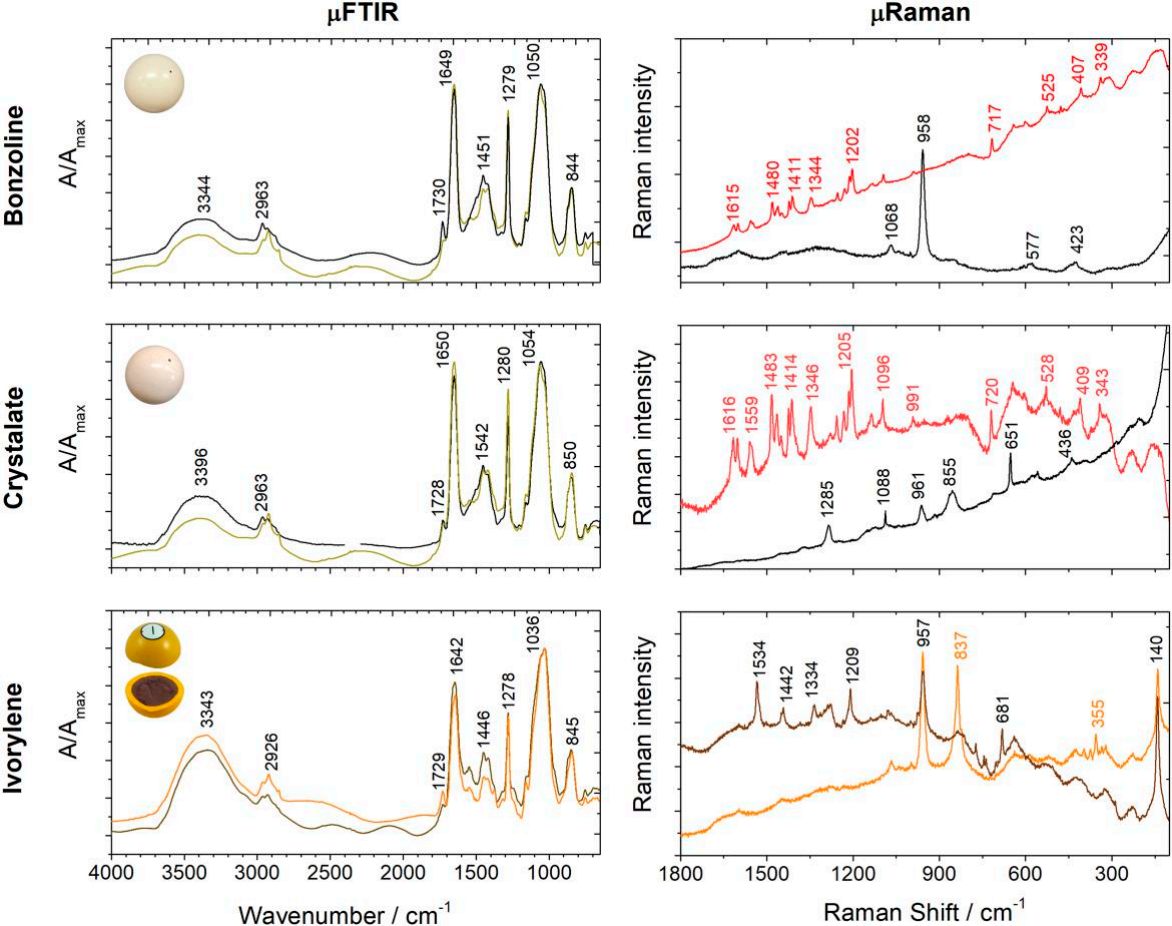
Molecular characterization of the bonzoline and crystalate billiard balls and ivorylene pool ball. The average infrared spectra obtained for the bonzoline and crystalate billiard balls (black spectra) are compared with the average spectrum obtained for the 1868 Hyatt billiard ball. The μRaman spectrum of white bonzoline billiard balls shows a characteristic spectrum of bone (black spectrum). White crystalate μRaman spectrum shows additional bands related to CN groups, calcite, camphor, and ZnO. Both red bonzoline and crystalate billiard balls show the characteristic bands of synthetic red pigment barium lithol red (PR49:1). The ivorylene infrared spectra of both the brown core (brown spectrum) and the yellow layer (orange spectrum) show the main absorptions of the bone–celluloid composite. The μRaman spectrum of the ivorylene yellow layer (orange spectrum) identified bone, chrome yellow, and anatase; and of the brown core sample (brown spectrum) phthalocyanine green (PG7) and anatase.

Hyatt's 1868 billiard ball showed an overall granular heterogeneous surface. With visible light microscopy, μFTIR, and SEM-EDS, it was disclosed that this billiard ball is composed of bone particles of several sizes on a celluloid matrix (Figs. 2 and 3). The 1868 billiard ball proportions of bone (B) to CN were quantified with μFTIR: 77% B to 23% CN (± 6% SD), which correlates with the formulation of USP 89582, 1869 and with the date of the billiard ball (Table 1, Figs. S2 and S3). In more detail, with SEM-EDS, it was possible to assess the heterogeneity of the mixture at the microscale: the phosphate regions show the variable dimensions of the bone particles (50 μm to submicrometer values); the detection of micro- and nanoparticles of aluminosilicate materials adds to the complexity of the mixture (Figs. 3 and S4). This was confirmed by the detection of ultramarine blue (Na_8–10_Al_6_Si_6_O_24_S_2–4_) particles by μRaman, used in low quantities likely to enhance the whiteness of the billiard ball (Fig. S5) (24). With XRF spectroscopy, it was possible to identify zinc and calcium as the main components (Fig. 4). Zinc is associated with the white pigment zinc oxide (ZnO), identified with μRaman by detecting its strong E_2_ vibration (Zn−O bending) at 436 cm^−1^, and calcium to calcite (CaCO_3_) by the ${\mathrm{CO}}_{3}^{2-}$ vibration at 1,087 cm^−1^ (Figs. 5, S6, and S7) (25, 26).

**Table 1.** Formulations of the 1868 Hyatt, ivorylene pool, bonzoline, and crystalate billiard balls.

	Ivory (1880–1920)	Hyatt (1868)	Ivorylene (1930s–1950s)		Bonzoline (1930s–1960s)	Crystalate (1900–1960s)	USP 89582 (1869)	USP 105338 (1870)
			Shell	Core				
% B to CN	—	77% (±6)	83% (±1)		80% (±2%)	79% (±2%)	75%	—
% C to %CN	—	13% (±1)	25% (±2%)		Surface: 25% (±0.7) Interior: 29% (±0.2)	19% (±1%)	—	33%
%B: %CN: %C^a^	—	74.8% B: 22.3% CN: 2.9% C	79.6% B: 16.3% CN: 4.1% C		75,9% B: 19.0% CN: 5.1% C	76.0% B 20.2% CN 3.8% C	—	—
Other additives	—	Aluminosilicates Calcium carbonate	Aluminosilicates Barium sulfate		Calcium carbonate		—	—
Main colorants^b^	—	Zinc oxide (Direct/American Process 1850s) (18) Ultramarine blue (1828) (19)	Chrome yellow PbCrO_4_ (1835) (20) Anatase, TiO_2_ (1926) (21)	Phthalocyanine green G (1938) (22) Anatase, TiO_2_ (1926)	Zinc oxide (1850s) *b*-naphthol red PR49:1 (1920s) (23)		—	—
Density g/cm^3^	1.73 and 1.80	1.92			1.93 ± 0.01	1.97 ± 0.01		

Weight proportions of bone (B) and camphor (C) are given in relation to CN as calculated by the calibration curves by infrared spectroscopy; for details, please see the supplementary material.

^a^The total is expressed as a percentage of each ingredient. Using patents USP 89582 (1869) and 105338 (1870), we can hypothesize the following percentages as an industrial formulation used by Hyatt: 69.3% bone: 23.1% celluloid nitrate: 7.6% camphor.

^b^For the dates of introduction in the US market of the synthetic pigments, please see references.

Peptide mass fingerprint (ZooMS) was used to identify the animal origin of the bone particles. By comparison with matrix-assisted laser desorption-ionization time of flight mass spectrometric (MALDI) marker ions from known references, it was found that the collagen from Hyatt's 1868 billiard ball is from cattle, indicating that the material used was probably bone dust, a by-product of the cattle industry (Fig. S8).

### Bonzoline (1930s) and crystalate (1900–1960s)

The characterization of the white bonzoline and crystalate billiard ball showed consistency with the composition found in the Hyatt 1868 billiard ball (Fig. 5). For bonzoline, with μFTIR, the proportion of bone to CN was quantified as 80/20% by weight (±2% SD), and the origin of the bone was confirmed to be cattle by ZooMS (Table 1, Figs. S9 and S10). For crystalate, the proportion of bone to CN was quantified as 79/21% by weight (± 3% SD; Table 1, Fig. S11). The XRF spectra of the two materials were similar, showing the intense emission lines of zinc and calcium (Fig. 4). This correlated with the μRaman spectra, which showed the presence of ZnO, by the observation of the Zn−O bending vibration at 436 cm^−1^, and calcite (CaCO_3_) by the ${\mathrm{CO}}_{3}^{2-}$ vibration at 1,087 cm^−1^ (Figs. 5 and S12).

Bonzoline, crystalate, and Hyatt's 1868 billiard balls showed different concentrations of camphor. The patent for the bone–CN composite of 1869 May 4 (89582) did not mention camphor (Table S1). Hyatt patented camphor (C) as a plasticizer for CN in 1870 (USP 105338), the fundamental patent for celluloid, in a proportion of 33% C to 77% CN. The ratio by weight of camphor to CN was quantified with μFTIR for the three billiard balls (Table 1): Hyatt's 1868 billiard ball has a proportion of 13% C to 87% CN (±1% SD, surface; Fig. S13); bonzoline 25–29% C to 75–71% CN (lower concentrations at the surface compared with the interior; Fig. S9); and crystalate a proportion of 19% C to 81% CN (±1% SD, surface; Fig. S11). The lower concentration of camphor in the Hyatt's 1868 billiard ball can be related to degradation, i.e. volatilization of camphor or due to a higher amount used in bonzoline/crystalate (27).

Remarkably, the same synthetic pigment was identified for the red bonzoline and crystalate balls: a β-naphthol red, possibly the pigment lake PR49:1 (lithol red having Ba^2+^ as a metal ion pair), part of a family that includes some of the most stable synthetic reds (23). The presence of barium was confirmed with XRF (Fig. 4). PR49:1 was identified with μRaman by the vibration bands at 1,615, 1,599, 1,455, 1,480, 1,461, 1,447, 1,422, 1,411, 1,344, 1,253, 1,229, 1,212, 1,202, 1,094, 717, and 525 cm^−1^, which correlated with a reference spectrum and with literature reference values (Figs. 5 and S14) (28).

We measured the densities of two NMAH's ivory billiard balls (1.73 and 1.80 g/cm^3^), the Hyatt's 1868 billiard ball (1.93 g/cm^3^), bonzoline (1.92 g/cm^3^), and crystalate (1.97 g/cm^3^; Table 1). We also measured the micro-Vickers hardness of a white bonzoline ball and obtained an average value of 22 HV (±3, 10 measurements). This value falls between the Vickers hardness values given by Vollrath et al. (29) for bone (30 HV) and celluloid (12 HV), which correlates with the chemical characterization of the bone–celluloid composite.

### Ivorylene pool ball

**Fig. 6.**
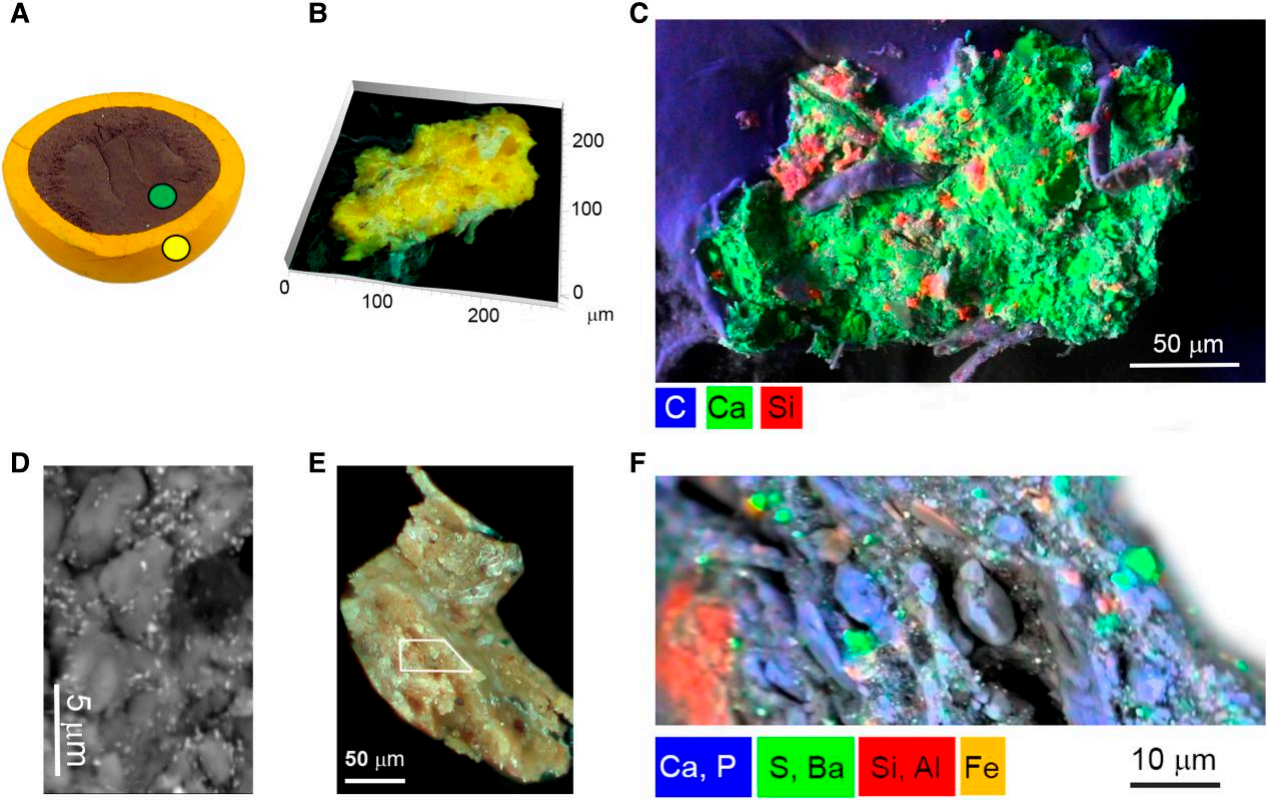
Microimaging of the ivorylene pool ball. A) Location of the samples analyzed: the brown core and the yellow layer. B) Yellow layer sample viewed using 3D visible light microscopy. C) Composite SEM-EDS image showing the distribution of carbon (blue), calcium (green), and silicon (red). D) Backscattered electron image showing submicrometer-sized chrome yellow particles, white particles within on a gray matrix made of complex CN:bone:camphor and other additives shown in C). E) Brown core sample under the visible light microscope. The white polygon represents the area imaged. F) Distribution of calcium (Ca) and phosphorous (P) in blue for bone, iron (Fe) in orange, aluminum (Al), and silicon (Si) in red for aluminosilicates, barium (Ba) and sulfate (S) in green for the additive barium sulfate, BaSO_4_; by SEM-EDS.

Remarkably, both the yellow shell and the brown core of the ivorylene pool ball are made of the bone–celluloid composite, with the overall weight proportions of 83% bone to 17% CN, by μFTIR (Figs. 5 and S15). By ZooMS, the bone origin was again identified as cattle (Fig. S16). In agreement with XRF data, which displays intense X-ray emission lines of lead and chromium, the coloring agents of the yellow exterior section were identified by Raman spectroscopy as chrome yellow (lead chromate, PbCrO_4_) and anatase (TiO_2_) (Figs. 4 and 5 and S17 and S18); SEM-EDS imaging of a yellow microsample showed an overall distribution of calcium and phosphorous from bone, silicate particles, and a type of organic fiber in which anatase was identified (Fig. 6). The fibers are likely cotton based on carbon and oxygen X-ray intensities. Hyatt's pre-1870 patents mention using paper in the filler for billiard balls (Table S1). Thus, it is possible that titanium white was not used as a pigment but was present due to the paper used in the filler (30). Electron imaging revealed that the mean diameter of the lead chromate particles was 204 nm ± 36 nm (*n* = 10), a value that is consistent with the intense pigmentation and opacity of lead chromates (Fig. 6) (31).

In the brown core, it was possible to observe a higher intensity of iron (Fe) X-ray emission lines (Fig. 4). This agreed with the detection by SEM-EDS of Fe aluminosilicates and other Si-Al-Mg-K-rich particles dispersed in the bone–celluloid matrix, which indicates the presence of clay minerals that correlate with the brownish color of the core (Figs. 6 and S19) (32). The other coloring agents found in the core were anatase (TiO_2_), phthalocyanine green G (PG7), and the filler barium sulfate (BaSO_4_; Figs. 5 and S20). XRF also detected lead (Pb) and indicated the presence of chrome yellow, but copper (Cu), an essential component of phthalocyanine green G, was not detected by XRF (Fig. 4). These findings agree with the economic claims of patent USP 259984 (1882): in 1931, the price of chrome yellow was 15 cents per pound, while clay and barite (BaSO_4_) were sold at circa 1 cent per pound (33, 34). It is also important to consider that a complete set of 16 pool balls has 8 colors. Although pigments are usually present in small amounts, different pigments have different densities. For example, chrome yellow has a density of ∼6.3 g/cm^3^, anatase ∼3.9 g/cm^3^, and barite ∼4.5 g/cm^3^. As a result, constructing a set of pool balls with an identical core diameter would mitigate the weight differences imparted by the differing materials in the exterior shell. However, explaining the presence of PG7 in a core meant to be unseen is a challenge.

## Discussion

### An unprecedented polymer composite: the contribution of heritage science to plastic material culture

By concentrating on a significant class of artifacts within the history of plastics—billiard balls—this work demonstrates the advantages of interdisciplinary studies between historians and heritage scientists. The accurate interpretation of the Smithsonian Institution's Hyatt's 1868 “original” billiard ball, and of the material that composes it, was only achieved by integrating the chemical characterization of billiard balls with a thorough investigation of historical sources on the late 19th early 20th century billiards world.

All billiard balls analyzed were consistently made of a composite material of ground bone, CN, and camphor. The ratio of ground bone to CN was quantified by μFTIR and correlated with a Hyatt's patent of 1869 May 4 (USP 89582), where the inventor mixed these two materials in a ratio of 75/25% to make an “ivory imitation.” The date of this patent correlates with the date of Hyatt's 1868 “original” billiard ball, indicating that this billiard ball was the prototype for a successful billiard ball industry. Billiard balls made with Hyatt's composite, or, as we propose it to be called, reinforced celluloid, were an alternative to ivory and were used for almost 90 years. As such, they represent a premier example of a reinforced polymer composite, one of the most widespread materials in science and technology today.

These results highlight that celluloid has been disregarded as a successful billiard ball because we have focused our historical research almost exclusively on the development of polymers and their uses in unalloyed forms. Celluloid and reinforced celluloid exhibit completely different properties. The enormous creativity of Hyatt and his co-workers in reinforcing a CN–camphor matrix with ground bone and producing a ball from this material was no small thing. This example emphasizes the need for a more comprehensive understanding of the 19th century inventors’ technological problem-solving strategies.

As the effort for ivory replication continues, the challenges remaining and those successfully met are worth noting. The problem of the global ivory trade and its impact on the African elephant population is still a critical issue in wildlife conservation (35). Modern material scientists have referred to ivory as a “model bio-composite.” One approach to developing synthetic ivory involves the combination of organic and mineral components in bio-inspired ratios (29). The early 75/25% wt. ground bone–CN ratio developed by Hyatt in 1868 is noteworthy, given its significant role in diminishing demand for ivory in an important market.

### The impact of reinforced celluloid in billiards

With the elucidation of the compositions of bonzoline, crystalate, and ivorylene, it becomes possible to conduct an examination of the impact of reinforced celluloid in the context of billiards, while gaining insights into the factors underlying its success. After Hyatt's invention, many billiard professional players continued to believe that ivory was better than the artificial alternative. One of the reasons professional players continued to play with ivory was related to prejudice. In 1899, John Roberts Jr. (1847–1919) played a game against Charles Dawson (1866–1921), where these two famous British champions argued about the type of balls to use, bonzoline or ivory. Dawson wanted to play with ivory mainly because of its socioeconomic status: “whoever heard of a money match of any importance being played with Bonzoline balls?” (11). There are different possible reasons why John Roberts Jr. wanted to play with bonzoline. The first has to do with marketing. Dawson accused Robert Jr. of having a “pecuniary interest in playing with Bonzoline billiard balls.” It was a common practice for manufacturers to contract famous players to bring their products to notice and prestige (Figs. S23 and S24) (16). For example, in 1904, Dawson (who lost against Roberts Jr. in 1899) was endorsing the Bonzoline Manufacturing Company, London. In the same year, H. W. Stevenson, a rival English billiards champion, promoted the competitor, the crystalate balls manufactured by Endolithic Manufacturing Co., London.

This work provides insights on the importance of uniform density. Density and hardness were two fundamental properties that had been standardized by ivory. As shown in this work, the densities of ivory and Hyatt's billiard balls were different: two ivory billiard balls varied from 1.73 to 1.80 g/cm^3^, while Hyatt's 1868 and bonzoline showed higher but remarkably consistent densities, considering their age difference of 60 years or more, between 1.92 and 1.93 g/cm^3^. The average density of the crystalate was even higher, 1.97 g/cm^3^, but the standard deviations for each three-ball set made of crystalate, and bonzoline, were very low, <0.01 g/cm^3^ (Table 1). Different billiard ball weights influence the player's approach to the game by the mechanics of the ball itself or, for example, by the selection of an appropriate cue stick (36). This was well understood in the early 20th century, as illustrated by relevant sources on cue sports that discussed the mechanical impacts of playing with these different materials since the “composition” billiard balls were “heavier” (12, 13, 37).

Uniformity, consistency, and reproducibility are crucial factors in modern sports equipment development. Uniformity in material properties ensures that athletes can rely on the same level of performance in every game or competition (38, 39). The density of elephant ivory is not uniform; it can vary from 1.7 to 1.9 g/cm^3^, depending on elephant and tusk characteristics (40). Furthermore, manufacturing perfectly balanced ivory billiard balls was a very complex process that required skilled workers (41). Sources on cue sports mentioned variations in the ivory billiard balls’ properties from set to set (42, 43). Due to these variations, it was a common practice to measure the ivory billiard balls’ sizes and weights in the conspicuous presence of the spectators prior to important matches (17). The invention of an artificial composite material allowed the manufacturer to control the properties of the billiard ball. The advantage of reinforced celluloid is described in historical sources that mention improved performances using composition billiard balls due to their “better” properties, such as Roberts Jr., H. W. Stevenson, Willie Smith (1886–1982), or Tom Newman (1894–1943) (10, 11, 13). As such, due to its innovative characteristics, reinforced celluloid contributed to the modern idea that artificial materials improve on the natural world and allow athletes to break records. Today, billiard balls, uniformly made of phenolic resins, are not weighed prior to the matches, suggesting the impact of reinforced celluloid in that regard.

Another problem with ivory was its susceptibility to fluctuations in relative humidity, leading to deformation and cracking (44). Players were advised on the adverse effects of weather on ivory billiard balls (3, 45–47). Bonzoline was marketed as superior to ivory in tropical climates (Fig. S21). J. Roberts Jr. played in India regularly, where he probably started using bonzoline (10). Comparing the yearly variations in relative humidity between Mumbai, a tropical climate where Roberts had a billiard table business, and London, a temperate climate, the former exhibits more prominent fluctuations (Fig. S22). Other factors related to geography and colonialism also appear relevant in material choices. For example, Walter Lindrum (1898–1960), an Australian billiards prodigy playing with composition billiard balls, was invited to play in Great Britain as British professionals continued to use ivory partly because they felt confident in beating the colonial challengers with this natural material (10).

The material choice made by amateur players was straightforward; it depended on function and economics. The composition billiard balls performed very well and were cheaper. In 1885, B-B-C sold a set of 4 standard-size Hyatt billiard balls for $10.50, while the ivory set cost $22 (the 2023 equivalent costs are $325.65 and 682.32, respectively), around 50% of the price of ivory as required by Phelan & Collender challenge in 1864 (Fig. S25). The technical and economic advantages of Hyatt's composite enticed the growth of pool and snooker, which required more balls. In 1928, B-B-C sold 16-ball ivory pool sets for $130285, while sets made of ivorylene were available at $20–25 ($2,315.01–5,075.21 versus $356.15–445.19 in 2023, respectively; Fig. S26). A new generation of players who learned to play with composition balls ultimately led to the complete transition from ivory to plastic (13). In 1926, the Billiard Association and the Control Council adopted crystalate balls for amateur championships. Finally, in 1929, it was pronounced that for the professional championship of English billiards, “all games to be played with Crystalate” (48).

## Future work

The discovery of reinforced celluloid and its success brings an entirely new set of questions. In 1883, billiard balls of pure celluloid (with no fillers) and bonsilate, another composite material reportedly developed by Hyatt and co-workers, were produced in Albany, New York (49). If a substitute for ivory billiard balls had been achieved as early as 1868, why did John Wesley Hyatt continue experimenting (and likely fail) with other compositions? Building on the methodology developed in this study, we will further investigate the diverse formulations of billiard balls and fully characterize their mechanical properties to understand the evolution of their development, manufacture, and use. This research will contribute to a deeper understanding of ivory consumption's social and environmental impact and the past efforts for its replacement. The pigments used to color the balls are fascinating and, on their own, bring us a chronology as they have been replaced over time. Ultimately, we can only interpret and preserve these significant historical objects with a complete study of their materiality.

This research is driven by paleo-inspiration, which uses historical systems and their eco-friendly production methods to drive innovative material design that targets specific mechanical, optical, or structural properties (50). Reinforced celluloid was made with plant-based materials and an animal by-product. Contemporary billiard balls are mainly produced from petroleum-based materials such as phenol-formaldehyde and polyester resins. Reinforced celluloid can become a useful “paleo-inspired” reverse-engineering model to develop innovative and sustainable materials for billiard balls. It is, however, essential to acknowledge that the history of celluloid exemplifies the consequences of uninformed exploration of renewable resources; the expansion of celluloid production in the 19th century led to an intensification of the camphor market, resulting in the decimation of Taiwan's forests and displacement of indigenous communities (51).

In the project “The Plastics Metamorphoses—the reality and the multiple approaches to a material” (Maria Elvira Callapez, PI, Robert Friedel, co-PI), we will deepen the multiscale analysis of reinforced celluloid historical systems. Correlations between the heterogenous microenvironments, processing methods and performance will be examined. The past processes for creating these complex systems will be reconstructed at a laboratory level to gain a thorough understanding of how these systems were developed. Reconstructions will be artificially aged to inform on how the microstructure and processing methods impact performance over time. Finally, the information gathered will allow the establishment of innovative and sustainable conservation strategies.

## Materials and methods

### Historical billiard balls

NMAH has 10 billiard balls identified in their records as CN or celluloid. The Celanese Corporation donated the Hyatt's 1868 billiard ball, studied in this work (ID number CH.334311, diameter 57 mm, 187 g). The ivorylene billiard ball studied (ID number 1997.0071.08, 57 mm) is part of an 11 billiard ball collection donated by the Albany International, former Albany Billiard Ball Company, in 1997. The interest in analyzing 1997.0071.08 came from the fact that it was already broken in halves and allowed the in situ analysis of its interior. The bonzoline (48 mm, 111.2g ± 0.5) and crystalate (41.5 mm, 73.2 g ± 0.2) billiard balls were acquired for analysis. Both sets are composed of three balls, two whites and one red, enclosed in their original box (Figs. S21 and S27). The bonzoline box mentions “made in England.” Bonzoline started being manufactured in England in 1931 by the Composition Billiard Ball Supply Co., London. Crystalate billiard balls were manufactured from 1909 until the development of Super Crystalate in 1972, a phenol-formaldehyde-based material. For comparison with the ivorylene billiard ball, one of the bonzoline billiard balls was cut in half, using a regular saw and polished with a Micro-Mesh wet sanding cloth grit 8000. The densities of two NMAH's ivory billiards balls were measured (ID Number CL.329507) 54 mm and 143.8g and 57 mm and 174.2 g (Fig. S28).

### XRF spectroscopy

A portable XRF spectrometer S1 Titan from Bruker was used on-site with the following experimental parameters: 40 kV, 6 μA and acquisition times between 5 and 20 s. The micro-XRF of the bonzoline and crystalate billiard balls was acquired on a Bruker ArtTAX Pro spectrometer equipped with a Molybdenum (Mo) ampule, Peltier effect cooled Xflash 3001 semiconductor detector, and a movable arm. Experimental parameters: 40 kV, 300 μA, 200 s, and helium atmosphere. Elements were identified by their characteristic X-ray emission lines using ARTAX software.

### 3D light microscopy and SEM-EDS

NMAH microsamples (Hyatt's 1868 and ivorylene) were analyzed with focal stack visible light microscopy using a Hirox KH-8700 digital microscope. The compositional distribution of major and minor elements was collected at the micrometer to submicrometer scale using a Hitachi SEM and a Bruker XFlash 6|60 EDS detector. The detailed description of the method is described elsewhere (52).

### Micro-Fourier transformed infrared spectroscopy

Infrared spectra were acquired on a Nicolet iS50 FTIR spectrophotometer and a Nicolet Nexus spectrophotometer, both equipped with a Nicolet Continuμm (15× objective) microscope and a Mercury–Cadmium–Tellurium (MCT) detector cooled by liquid nitrogen. Microsamples were placed between diamond cells for sample compression, and the spectra were acquired in transmission mode between 4,000 and 650 cm^−1^, with a resolution of 8 cm^−1^ and 128 scans. Microsamples were collected using Ted Pella μ-tools an M8 Wild Heerbruug stereomicroscope (6–50× magnification) and a Leica MZ16 stereomicroscope (between 7.1× and 115×).

The quantification of the formulations was achieved by the development of calibration curves using the absorbance ratio method, i.e. by mixing two components in different weight proportions: (i) for the quantification of the proportion of CN to ground bone and (ii) for the quantification of camphor to CN. No animal participant was involved in this study. The cattle (*Bos taurus*) ground bone was prepared by grinding a slaughterhouse waste femur bone provided by a local butcher. The comprehensive details are given in Figs. S2, S3, and S13.

### Raman spectroscopy

Raman MIRA DS is a handheld Raman spectrometer (Metrohm), operating with a diode laser (785 nm, 100 mW) and a 200–2,300 nm spectral range. The equipment provides a spectral resolution of 8–10 cm^−1^ and a spot size between 0.04 and 2.5 mm. The detection technique is Orbital Raster Scan, which scans a large area of the sample surface with a tightly focused beam that maintains a high spectral resolution, an advantage for heterogeneous materials without putting the material surface at risk. μRaman spectra were collected on a Labram 300 Jobin Yvon spectrometer equipped with a He–Ne laser (632.8 nm, 17 mW) and a diode laser (785 nm, 100 mW). The laser beam was focused with an Olympus 100× lens with a spot size of 2 μm. The conditions used are provided for each spectrum.

### Peptide mass fingerprint (ZooMS)

Peptide mass fingerprint analysis involves the enzymatic digestion of proteins followed by MALDI analysis of the resultant peptide mixture. Each protein has a unique sequence of amino acids, and thus, the mixture of peptides is unique—a “peptide mass fingerprint.” Marker ions in the MALDI spectra from known reference materials are compared with those from unknown samples for identification. The detailed description of the method is described elsewhere (53). This analysis was performed to gain information on the origin of the bone found in the billiard balls. If the billiard balls contained ivory dust, ZooMS should have found elephant markers, or other ivory animal sources such as walrus, narwhal, hippopotamus, warthog, sperm whale, or orca (35). ZooMS was the chosen method because of the small quantity of sample available (μg).

### Vickers hardness tests and density measurements

The micro-Vickers hardness of the bonzoline white ball was measured with a ZwickRoell ZHVμ micro-Vickers hardness tester, using a test force of 100 gf, and performing 10 measurements in different areas. Density was measured considering that the billiard ball is a perfect sphere, mass/volume.

## Supplementary Material

pgad360_Supplementary_Data

## Data Availability

All data are included in the manuscript and/or supporting information.

## References

[1] Phellan & Collender . 1864. Ten thousand dollars for a substitute for ivory. Sci Am. 10(11):166.

[2] Friedel R . 1983. Pioneer plastic: the making and selling of celluloid. Madison (WI): The University of Wisconsin Press.

[3] Shamos MI . 1993. The illustrated encyclopedia of billiards. New York (NY): Lyons & Burford.

[4] Palucka T . 2005. Artificial billiard balls. MRS Bull. 30:614.

[5] Hyatt JW . 1914. Address of acceptance. Ind Eng Chem. 6:168–170.

[6] Meikle JL . 1997. American plastic: a cultural history. New Brunswick (NJ): Rutgers University Press.

[7] Morris PJ . 1986. Polymer pioneers: a popular history of the science and technology of large molecules. Philadelphia: Chemical Heritage Foundation.

[8] Mossman S . 1997. Early plastics: perspectives 1850–1950. London and Washington: Leicester University Press and Science Museum.

[9] Rasmussen SC . 2021. From parkesine to celluloid: the birth of organic plastics. Angew Chem. 133:8090–8094. 10.1002/anie.202015095 33576547

[10] Everton C . 1986. The history of snooker and billiards. Colchester: TBS The Book Service Ltd.

[11] Roberts J, Hotine FM. 1902. Modern billiards. London: C. Arthur Pearson Ltd.

[12] Levi R . 1916. Billiards: the stokes of the game. Part III. Manchester: Riso Levi.

[13] Levi R . 1931. Billiards in the 20th century. Manchester: Riso Levi.

[14] Broadfoot MW . 1896. Billiards. London and Mumbai: Longmans and Green and Co.

[15] Reece T, Clifford WG. 1915. Billiards. London: A. & C. Black, Ltd.

[16] Mannock JP . 1904. Billiards expounded to al degrees of amateur players. London: Grant Richards.

[17] Clare N . 1988. Die Geschichte des Billard- und Snookerspiels. Billard (15):27.

[18] Osmond G, et al 2019. Zinc soaps: an overview of zinc oxide reactivity and consequences of soap formation in oil-based paintings. In: Casadio F, editor. Metal soaps in art. Cham: Springer. p. 25–46.

[19] Osticioli I, et al 2009. Analysis of natural and artificial ultramarine blue pigments using laser induced breakdown and pulsed Raman spectroscopy, statistical analysis and light microscopy. Spectrochim Acta A Mol Biomol Spectrosc. 73:525–531. 19129003 10.1016/j.saa.2008.11.028

[20] Otero V, et al 2016. Nineteenth century chrome yellow and chrome deep from Winsor & Newton™. Stud Conserv. 62:123–149.

[21] Brown S, Clark RJH. 2013. Anatase: important industrial white pigment and date-marker for artwork. Spectrochim Acta A Mol Biomol Spectrosc. 110:78–80. 23557776 10.1016/j.saa.2013.03.041

[22] Chaplin TD, Clark RJH. 2016. Identification by Raman microscopy of anachronistic pigments on a purported Chagall nude: conservation consequences. Appl Phys A. 122:144–148.

[23] Angelin EM, Oliveira MC, Nevin A, Picollo M, Melo MJ. 2021. To be or not to be an azo pigment: chemistry for the preservation of historical β-naphthol reds in cultural heritage. Dyes Pigm. 190:109244.

[24] Buxbaum G, Pfaff G. 2005. Industrial inorganic pigments. 3rd ed. Weinheim: WILEY-VCH Verlag GmbH & Co KGaA.

[25] Damen TC, Porto SPS, Tell B. 1966. Raman effect in zinc oxide. Phys Rev. 142:570.

[26] Sun J, Wu Z, Cheng H, Zhang Z, Frost RL. 2014. A Raman spectroscopic comparison of calcite and dolomite. Spectrochim Acta A Mol Biomol Spectrosc. 117:158–162. 23988531 10.1016/j.saa.2013.08.014

[27] Selwitz C . 1988. Cellulose nitrate in conservation. Los Angeles (CA): J. Paul Getty Trust.

[28] Vandenabeele P, Moens L, Edwards HGM, Dams R. 2000. Raman spectroscopic database of azo pigments and application to modern art studies. J Raman Spectrosc. 31:509–517.

[29] Vollrath F, Ruixin M, Shah DU. 2018. Ivory as an important model bio-composite. Curator (N Y). 61:95–110.

[30] Quye A . 2014. Factors influencing the stability of man-made fibers: a retrospective view for historical textiles. Polym Degrad Stab. 107:210–218.

[31] Erkens LJH, Hamers H, Hermans RJM, Claeys E, Bijnens M. 2001. Lead chromates: a review of the state of the art in 2000. Surf Coat Int Part B: Coat Int. 84:169–176.

[32] Velde B . 1995. Composition and mineralogy of clay minerals. In: Velde B, editor. Origin and mineralogy of clays. Berlin: Springer-Verlag. p. 8–42.

[33] Kiessling OE . 1933. Minerals yearbook 1932–33. Washington (DC): United States Government Printing Office.

[34] Kiessling OE . 1933. Mineral resources of the United States 1930. Washington (DC): United States Government Printing Office.

[35] Baker B, Jacobs R, Mann MJ, Espinoza E, Grein G. 2020. CITES identification guide for ivory and ivory substitutes. 4th ed. Washington (DC): World Wildlife Fund Inc.

[36] Alciatore D . 2004. The illustrated principles of pool and billiards. New York (NY): Sterling Publishing Co.

[37] Western CCM . 1911. The practical science of billiards and it's pointer. London: Simpkin Marshall Hamilton Kent & Co Ltd.

[38] Subic A . 2019. Materials in sports equipment. Duxford: Woodhead Publishing.

[39] Easterling EA . 2012. Advanced materials for sports equipment: how advanced materials help optimize sporting performance and make sport safer. Dordrecht: Springer.

[40] Elder WH . 1970. Morphometry of elephant tusks. Zool Afr. 5:143–159.

[41] Maskell A . 1906. Ivory in commerce and in the arts. J Soc Arts. 54:1173–1190.

[42] Newman T . 1923. How to play billiards. London: Methuen & Co. Ltd.

[43] Levi R . 1920. Billiards: ivory billiard balls and the professional championship. Mercury. 3:3.

[44] Lafontaine RH, Wood PA. 1982. The stabilization of ivory against relative humidity fluctuations. Stud Conserv. 27:109–117.

[45] Bennett J . 1894. Billiards. London: Thos. De la rue & Co.

[46] Brunswick Corporation . 1909. A complete handbook of standard rules of all the prominent games of billiards and pool. New York (NY): The Brunswick-Balke-Collender Company.

[47] Garno B . 1908. Modern billiards: a complete textbook of the game, containing plain and practical instructions how to play and acquire skill at this scientific amusement. New York (NY): The Brunswick-Balke-Collender Company.

[48] The Billiards Association and Control Council: conditions for the professional championship of English billiards, 1929 . 1928. The Billiard Player.

[49] The billiard ball: its process or manufacture—a very peculiar industry. 1883. The Washington Post (1877–1922).

[50] Bertrand L, Gervais C, Masic A, Robbiola L. 2018. Paleo-inspired systems: durability, sustainability, and remarkable properties. Angew Chem Int Ed. 57:7288–7295. 10.1002/anie.201709303 29154403

[51] Altman R . 2021. The myth of historical bio-based plastics. Science. 373:47–49. 34210874 10.1126/science.abj1003

[52] Vicenzi EP, et al 2022. Major to trace element imaging and analysis of iron age glasses using stage scanning in the analytical dual beam microscope (tandem). Heritage Sci. 10:1–15.

[53] Kirby DP, Khandekar N, Arslanoglu J, Sutherland K. 2011. Protein identification in artworks by peptide mass fingerprinting. ICOM Committee for Conservation 16th Triennial Meeting, September 19–23, 2011, Lisbon, Portugal. Critério Artes Gráficas, Lda and ICOM Committee for Conservation.

