# Correction to: Best billiard ball in the 19th century: Composite materials made of celluloid and bone as substitutes for ivory

**DOI:** 10.1093/pnasnexus/pgad445

**Published:** 2023-12-21

This is a correction to: Artur Neves, Robert Friedel, Maria J Melo, Maria Elvira Callapez, Edward P Vicenzi, Thomas Lam, Best billiard ball in the 19th century: Composite materials made of celluloid and bone as substitutes for ivory, *PNAS Nexus*, Volume 2, Issue 11, November 2023, pgad360, https://doi.org/10.1093/pnasnexus/pgad360

In the originally published version of this manuscript, there is an error in Figure 3. One of the SEM-EDS single-element images published in the final version was wrongly labeled as potassium (K), when it should be labeled as Calcium (Ca). The image caption is correct. The correct version of Fig. 3 is as follows:

**Figure:**
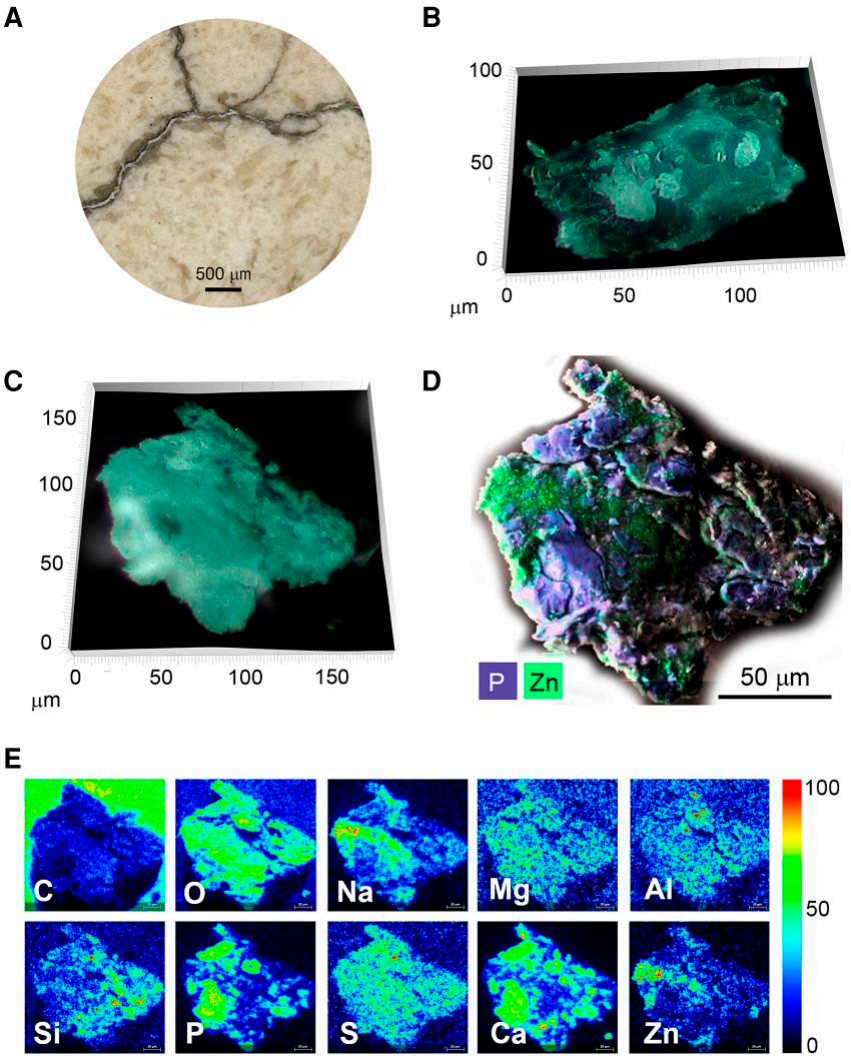


This error has been corrected online.

