# Supplementary material for: Best billiard ball in the 19th century: Composite materials made of celluloid and bone as substitutes for ivory
Source: PNAS Nexus. 2023 Nov 3;2(11):pgad360. doi: 10.1093/pnasnexus/pgad360 (PMC10651075; doi:10.1093/pnasnexus/pgad360)
Supplement: pgad360_Supplementary_Data [file pgad360_supplementary_data.docx]

**Supporting Information for**

Best billiard ball in the nineteenth century: composite materials made of celluloid and bone as substitutes for ivory

Artur Neves, Robert Friedel, M. J. Melo, Maria Elvira Callapez, Edward P. Vicenzi, Thomas Lam

*Artur Neves and Robert Friedel

Email: al.neves@campus.fct.unl.pt; friedel@umd.edu

**This PDF file includes:**

Supporting text

Figures S1 to S28

Tables S1

SI References

Supporting Information Text

Materials

For the construction of the infrared calibration curves were used the following materials: Bovine femur (Talhos Silau, Portugal), pure cellulose nitrate membranes (Amersham™Protran™ Cytiva), camphor (Sigma-Aldrich, 96%), methanol (Sigma-Aldrich, HPLC grade).

Bovine bone powder preparation

The cattle (Bos taurus) bone powder was prepared by grinding a fresh femur bone, supplied by a local butcher. In the first stage, the femur bone was hand sawed into smaller pieces (circa 5x2cm), using an 300mm standard metal saw (Dexter). The soft tissues were mechanically removed using a scalpel and a spoon and the bone cleaned in a surfactant solution of 0.1 wt.% in water (Teepol). To further reduce the particle size, the bone pieces were sawed again and hammered (<1cm). Finally, the bone was ground in an agate pestle and mortar for 20min (Retsch Mortar Grinder RM 200).

Preparation of bovine bone powder – cellulose nitrate samples

A solution of 2% w/w of cellulose nitrate in methanol was prepared at room temperature and allowing cellulose nitrate to dissolve through the night (approx. 12 hr). The bovine bone powder was mixed with this solution in different proportions by weight of bone and cellulose nitrate: 80%, 70%, 60% and 50% w/w bone to 20%, 30%, 40% and 50% w/w cellulose nitrate, respectively. The solution was stirred with a magnetic stirrer and the solvent allowed to evaporate until a homogenous dough was obtained (2-4h, higher times for higher amounts of cellulose nitrate/methanol). This dough was dried through the night (approx. 12h) in a desiccator with silica-gel. After drying, homogenous references of bone and cellulose nitrate were obtained.

Preparation of cellulose nitrate-camphor (celluloid) films

Cellulose nitrate-camphor (celluloid) films were obtained by adding camphor to a solution of 2% w/w cellulose nitrate in methanol. Camphor was added in different proportions by weight to cellulose nitrate: 10%, 20% and 35% w/w camphor to and 90%, 80% and 65% w/w cellulose nitrate, respectively. Camphor was allowed to dissolve through the night (approx. 12 hr). The solution was cast homogenously over the surface of a microscope glass slide using a Pasteur pipette. The microscope glass slides were placed inside a desiccator with silica-gel and the solution left drying through the night (approx. 12 hr). After drying, transparent cellulose nitrate-camphor (celluloid) films were obtained.

Construction of the bovine bone powder – cellulose nitrate μFTIR calibration curves

A bone micro-particle was collected from the 1868 Hyatt billiard ball, and the infrared spectrum acquired: it was possible to observe the strong PO_4_^3-^ vibration band at 1036 cm^-1^, the amide I (C=O stretching), amide II (mixed C-N stretching and N-H in plane bending) and amide III (mixed C-N stretching (18%-40%) and N-H in plane bending (40-60%) and additional contributions of C-C stretching) bands at 1644, 1551 and 1238 cm^-1^, respectively, the CO_3_^2-^ vibrations at 1451, 1418 (this doublet is due to B type substitution by CO_3_^2-^, the major substitution in bone and dentine) and 873 cm^-1^, in the region between 3600 and 3000 the amide A and amide B and OH streching, (Fig. S2). The carbonate content (CO_3_^2-:^PO_4_^2-^) and the mineral to matrix ratios (Amide I:PO_4_^2-^) of the reference and “original” billiard ball bone samples were measured with FTIR following the method by (1) (Fig. S2). The carbonate content is related to crystallinity and the mineral to matrix ration to the amount of collagen to the amount of hydroxyapatite. Both ratios decrease with the decrease of the particle size. For the cow bone reference, the CO_3_^2-:^PO_4_^2-^ was of 0.26 (±0.05, 3 spectra) and the Amide I:PO_4_^2-^ of 0.33 (±0.04, 3 spectra). For the original billiard ball, 0.22 (±0.04, 2 spectra) and 0.30 (±0.02, 2 spectra), respectively. These values evidence similar properties and particle sizes.

Infrared spectrum of cellulose nitrate was characterized by bending of the CH and CH_2_ bonds between 1500-1300 cm^-1^, respectively, the strong vibrations of the nitrate groups at 1651 (νsNO2), 1280 (νaNO_2_) and 842 (νNO) cm^-1^, and the cellulosic vibrational envelope, i.e., the stretching of the inter and intra ether bonds, between 1200 and 900 cm^−1^. The infrared spectrum of the bovine bone was characterized the amide I (1650 cm^-1^), amide II (1550 cm^-1^) and amide III (1240 cm^-1^) bands of collagen, the strong absorption of the apatitic phosphate of hydroxyapatite with maximum at 1036 cm^-1^ (νPO_4_^3-^), the ν3 and ν2 bands of the carbonate ions (CO_3_^2-^) in hydroxyapatite at 1451 and 873 cm^-1^. By mixing both materials, changes are more evident in the regions of strong absorption of the nitrate and phosphate groups. For the development of the calibration curve the ratio ν_a_NO_2_/νPO_4_^3-^ was plotted in function of the % w/w of cellulose nitrate in the mixture. The absorbances of both bands was measured using OMNIC software by applying a baseline correction. Baselines were delineated using the band height tool and the absorbance calculated from the maximum of the band to the corresponding baseline: for the ν_a_NO_2_, the baseline was delineated between 1800 and 1220 cm^-1^; for the νPO_4_^3-^, the baseline was delineated between 1185 and 925 cm^-1^. For each bovine bone powder-cellulose nitrate sample, 6 infrared spectra were collected and used. In fig. S2B, the average of the 6 infrared spectra for each sample and the absorbance measuring methodology is shown. A linear regression of the data was calculated using OriginPro 2016. The calibration curve obtained coefficient of determination (R^2^) of 0.983 (Fig. S3).

Construction of the cellulose nitrate-camphor μFTIR calibration curves

When mixed with cellulose nitrate, the influence of camphor in cellulose nitrate infrared spectrum is observed by the appearance of the carbonyl band (νC=O) at 1730 cm^-1^ and minor changes related to the vibrations of the CH bonds in and 1500-1330 cm^-1^. The relative absorbance of the carbonyl band increases with the increase of camphor concentration. For the development of the calibration curve, the ratio νC=O/ ν_a_NO_2_ was plotted in function of the % w/w of camphor in the mixture. The absorbances of both bands was measured using OMNIC software by applying a baseline correction. A baseline was delineated using the band height tool, between 1520 and 1220 cm^-1^, and the absorbance calculated from the maximum of the band to the corresponding baseline. For each cellulose nitrate-camphor reference sample, 3 infrared spectra were collected and used. The average of the 3 infrared spectra for each sample and the absorbance measuring methodology is shown. A linear regression of the data was calculated using OriginPro 2016. The calibration curve obtained had a coefficient of determination (R^2^) of 0.975 (Fig. S13)


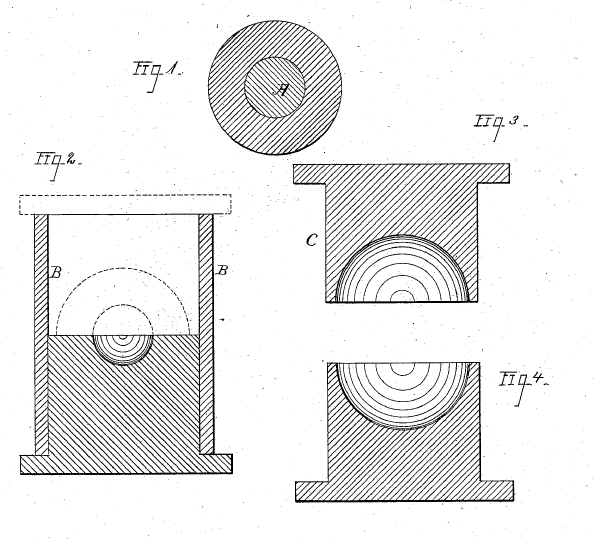


**Figure S1. Scheme of Hyatt’s US Patent 259984 of 1882.** Fig.1 is the central section of a billiard-ball made according to the invention, where A is the core and B is “a wall or shell adapted to fit over the core-set, the nature and construction of which is sufficiently illustrated in Fig. 2.”


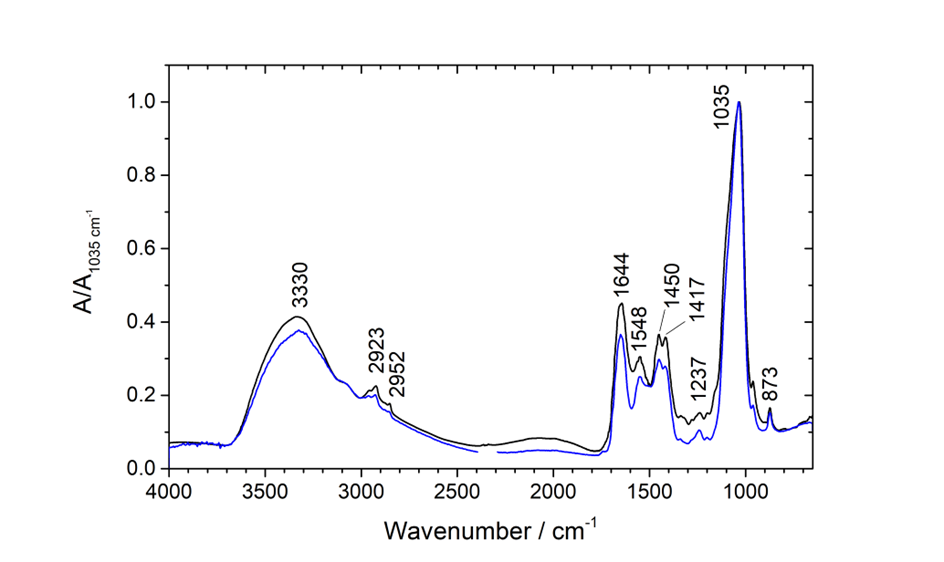
**Fig. S2.** Infrared spectra of a bone micro-particle collected from the 1868 billiard ball (black) and from the ground cow femur bone (blue).


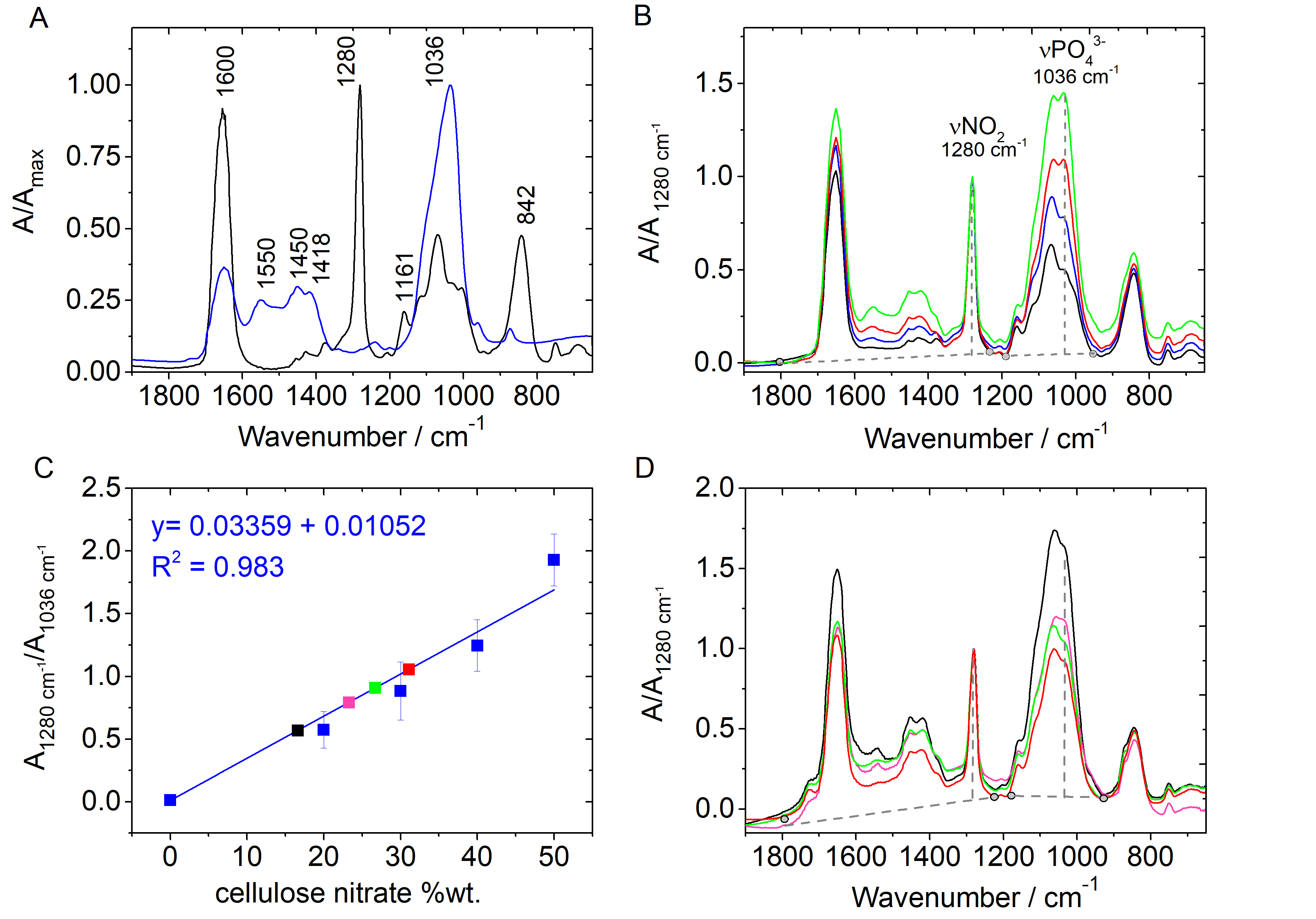


**Figure S3. Development of the bone-cellulose nitrate calibration line and quantification of the mixture in the 1868 billiard ball.** A) Infrared spectra of the ground cow bone femur (blue) and cellulose nitrate references (black) between 1900 and 650 cm^-1^; these materials were used for the development of the calibration curve. B) Overlay of the infrared spectra of the bovine bone-CN reference mixtures, normalized to the ν_s_NO_2_ (1280cm^-1^) band (%wt bone/cellulose nitrate): 80/20 (green), 70/ 30 (red), 60/40 (blue), 50/50% (black). C) The data shows the average ratio and the standard deviation calculated from 6 infrared spectra collected for each reference (blue). The cellulose nitrate concentrations calculated for 4 spectra of the 1868 billiard ball are showed (average 23%, standard deviation 6%). D) Overlay of the infrared spectra used for the quantification of the bone-celluloid mixture in the 1868 billiard ball. The colors correspond to the points in C. The spectra are normalized to the ν_s_NO_2_ (1280cm^-1^) band. Acquired from 4 different microsamples collected from the surface of the object.

**
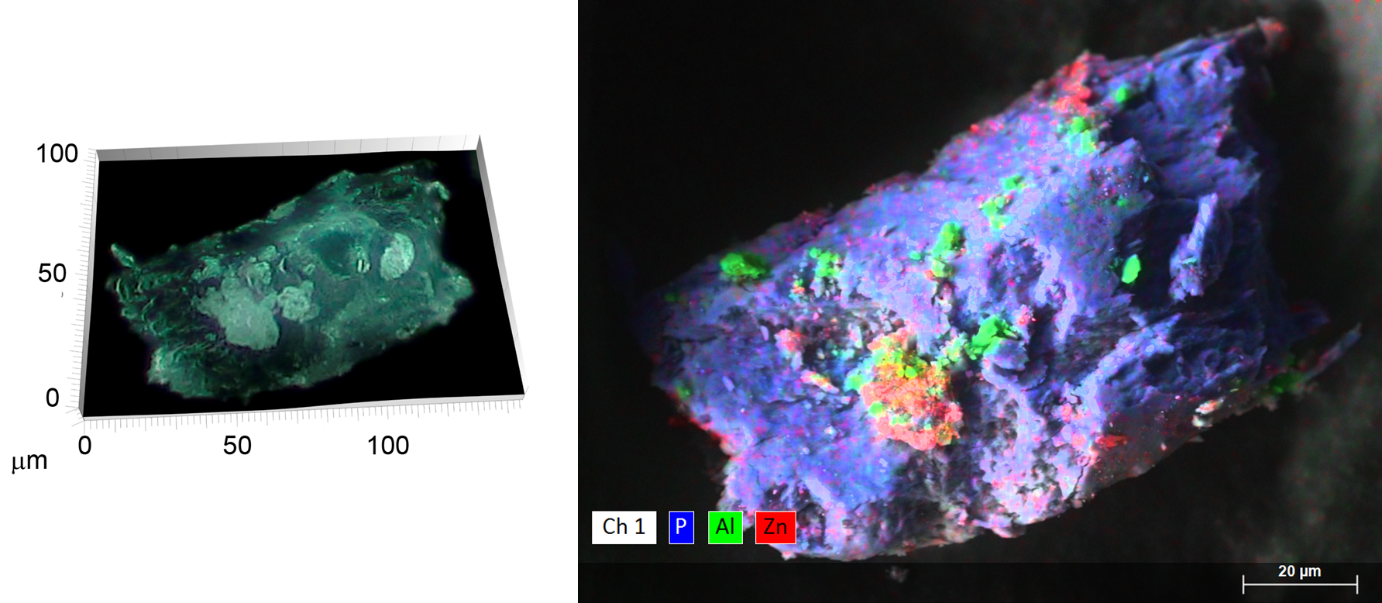
**

**Figure S4. SEM-EDS imaging of a bone particle acquired from the 1868 billiard ball sample.** The distribution of phosphorous (P), aluminum (Al) and zinc (Zn) is showed. It is possible to observe the homogenous distribution of phosphate in the bone matrix (Fig. S4). At the surface of the bone particle, aluminum (Al) particles were found, related to the presence of aluminosilicates. Furthermore, it was possible to observe a homogenous mass adhered to the sample of the bone particle attributed to presence of zinc (Zn), possibly from the celluloid zinc oxide mixture (Fig. S4).


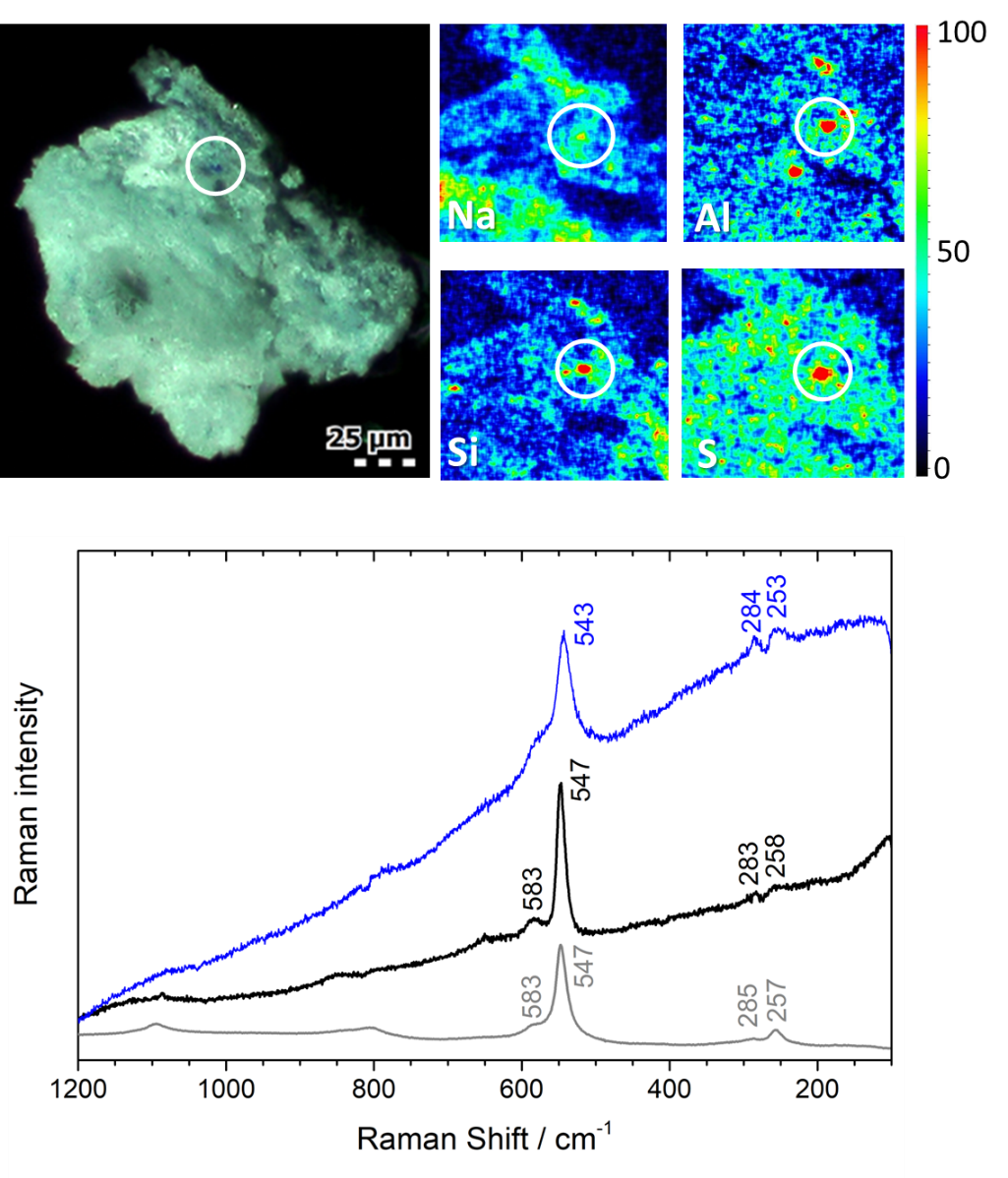


**Figure S5. SEM-EDS and Raman spectrum of the blue particle found in the 1868 hyatt billiard ball sample.** The SEM-ED component images show a zoom of the area where the blue particle was found, where it is possible to detect strong emission from sodium (Na), aluminium (Al), silicon (Si) and sulfur (S). The blue particle was analyzed with μRaman (blue spectrum, 785nm laser, 4.35mW, 20s, 10 cycles) and a strong band was observed at 543 cm^-1^. Another blue particle was found in a different microsample and analyzed with μRaman (black spectrum, 785nm laser, 4.35mW, 150s, 5 cycles). This sample was compressed providing a better-quality Raman spectrum, with the detection of a strong band at 547 cm^-1^. This band is attributed to the ultramarine blue’s symmetric stretching vibration (ν_1_) of S^3−^, as it is possible to compare with a reference Raman spectrum (grey, Casa Ferreira). Other bands were detected at 253-257 cm^-1^ attributed to the bending vibration (ν_2_) of S^3−^ and a unattributed shoulder at 583 cm^-1^.

**
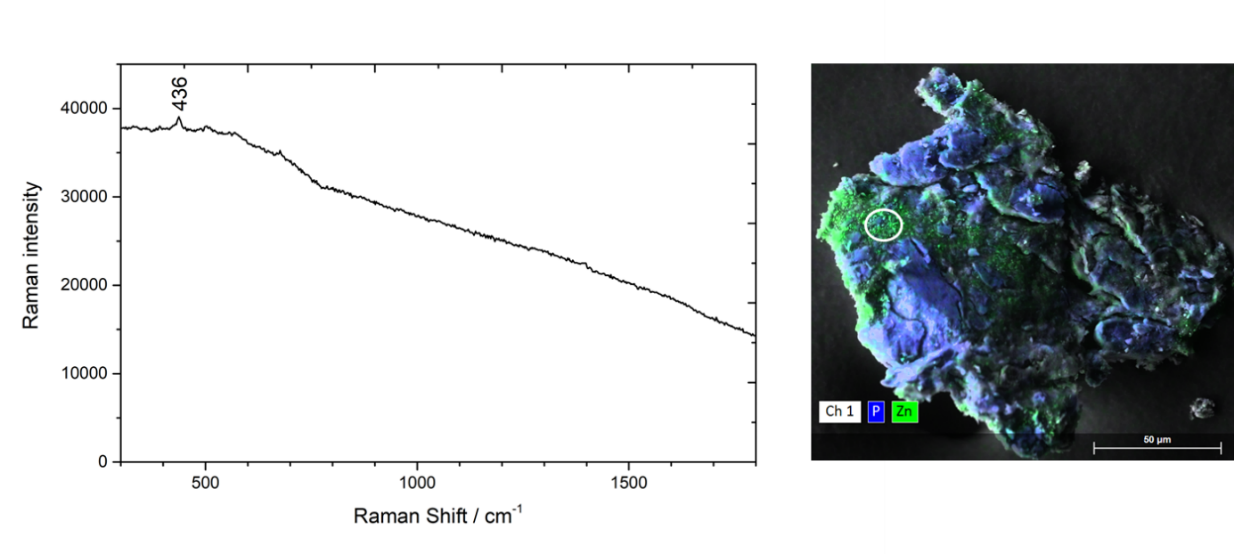
**

**Figure S6. μRaman spectra of the zinc particles (left) found in a microsample of the 1868 billiard ball imaged with SEM-EDS (right).** (633nm laser, 4.25 mW, 120s x 5 acquisition time). False color image of the SEM-EDS electron beam-induced X-ray imaging: phosphorous from hydroxyapatite in blue, zinc from ZnO in green. The region of μRaman analysis is marked by the white circle. Due to the fluorescence detected it was necessary to reduce the laser power and use longer acquisition times.


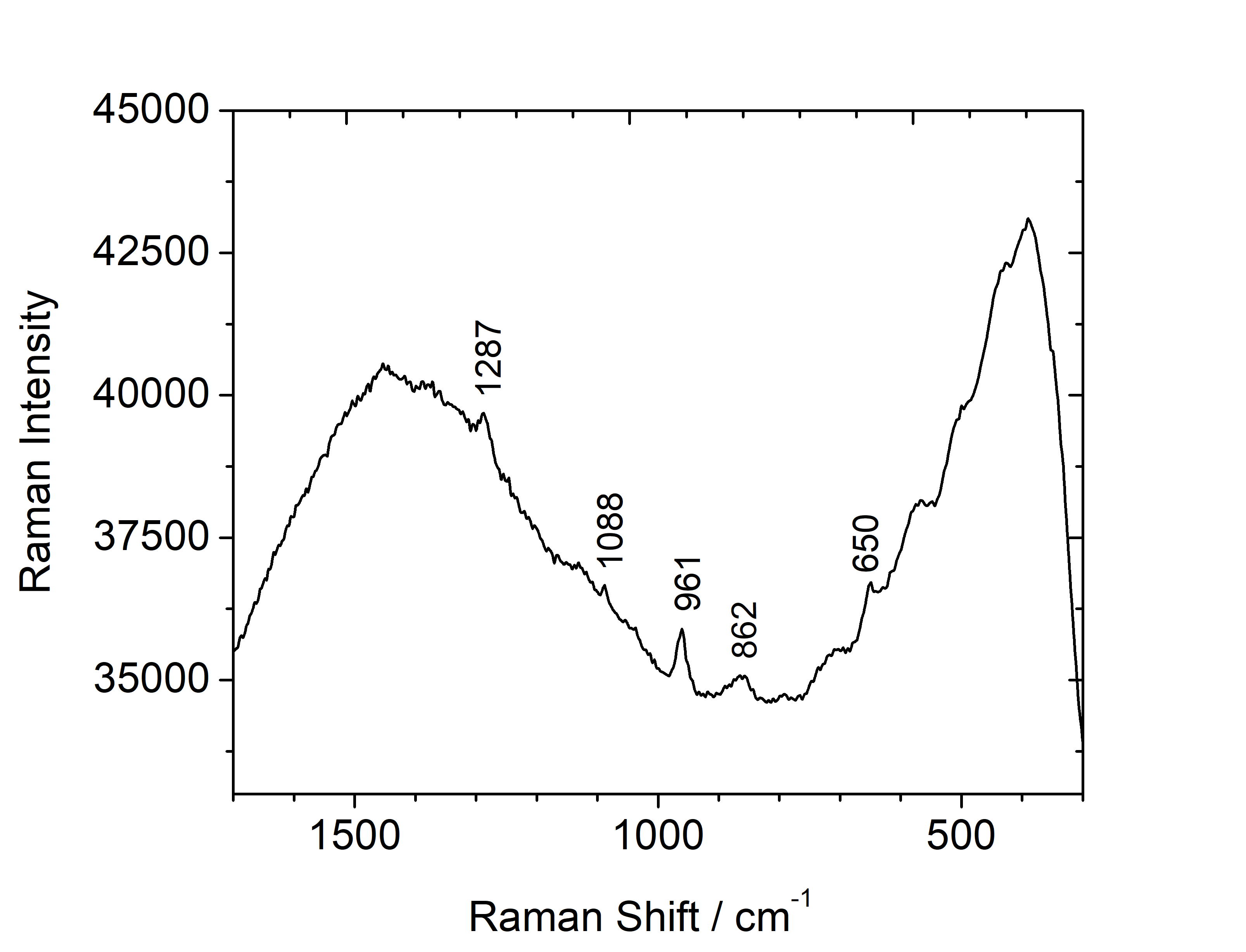


**Fig. S7.** Raman MIRA DS spectra obtained from the in-situ analysis of the 1868 billiard ball. Conditions: 1.20s acquisition time, 10 cycles. It was only possible to identify the most intense bands for each of bone, cellulose nitrate, and camphor. This is due to strong background noise. Since this effect was not observed when analyzing pure bone powder, and laser 785nm this does induce strong bone fluorescence this is probably due to another minor component in the mixture, such as a surface treatment, or due to degradation. Calcite (CaCO_3_) was also detected by the observation of the characteristic symmetric stretching of the carbonate (ν_s_CO_3_^2-^) at 1088 cm^-1^. This mineral carbonate does not exist in bone, meaning that it was intentionally admixed, possibly as chalk.


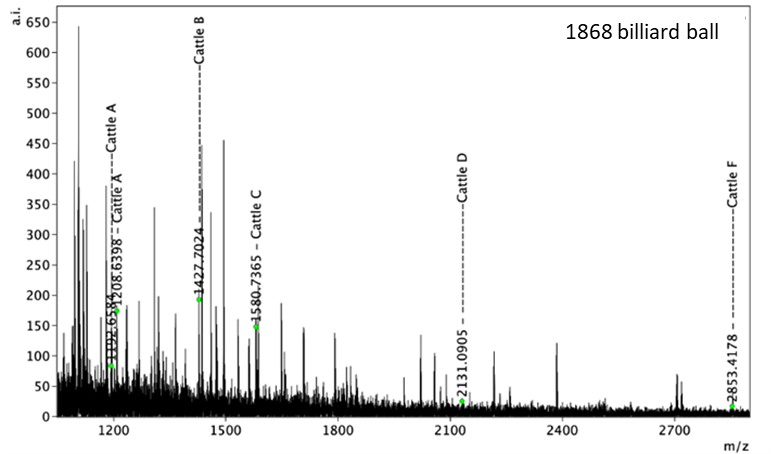


**Figure S8.** Peptide Mass Fingerprint MALDI spectra of the 1868 billiard ball. The cattle markers are indicated in the spectra.


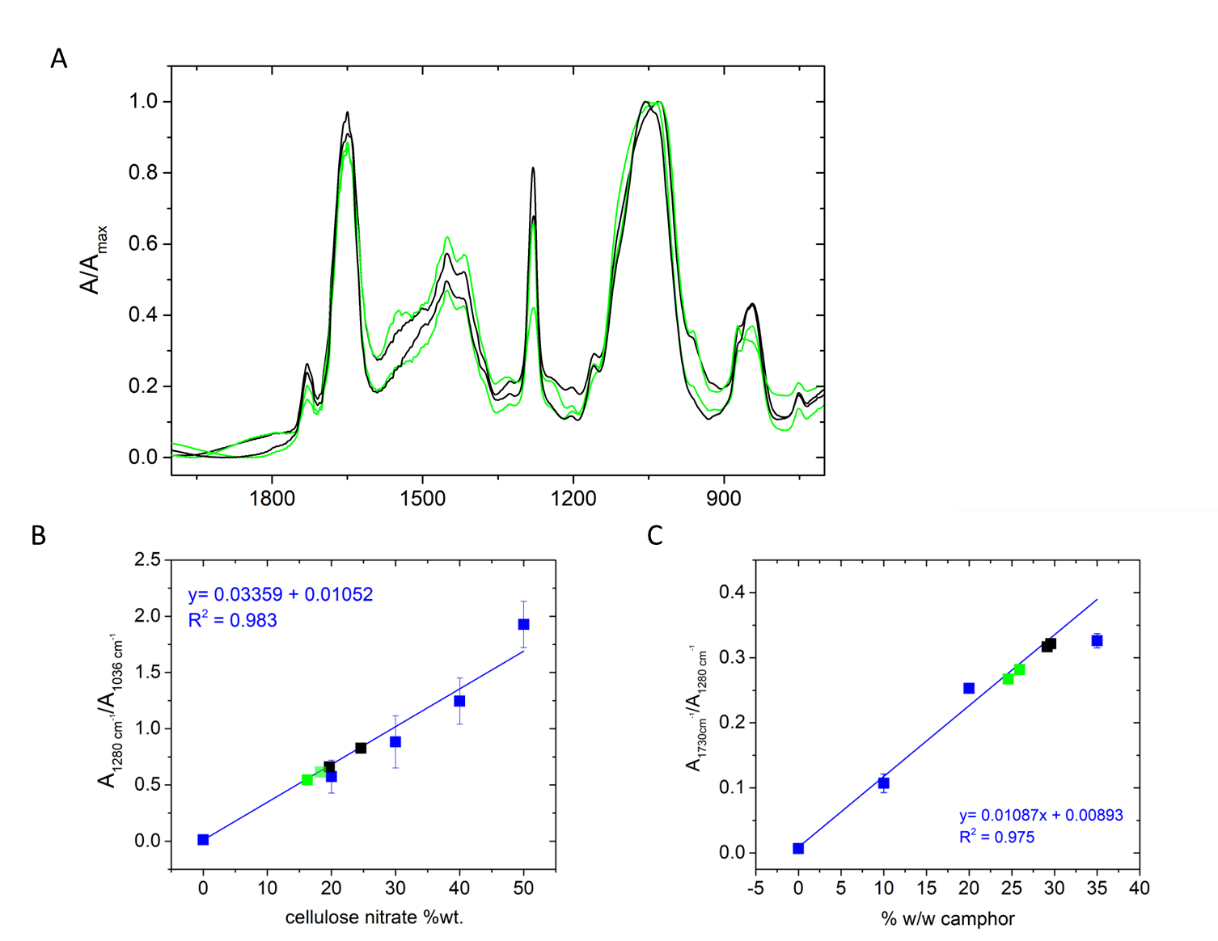


**Figure S9. Quantification of the bone-cellulose nitrate and camphor-cellulose nitrate formulation of the Bonzoline billiard ball.** A) Overlay of the infrared spectra of the four microsamples used for the quantification of Bonzoline formulation, 2 from the surface (green) and 2 from the interior (black). The spectra are normalized to the maximum (nPO_4_^2-^ (~1036 cm^-1^) band. C) The cellulose nitrate concentration calculated for the two regions are showed (average 20%, standard deviation 2%). D) The camphor concentrations calculated for the three regions are showed (average 27%, standard deviation 2%).

**
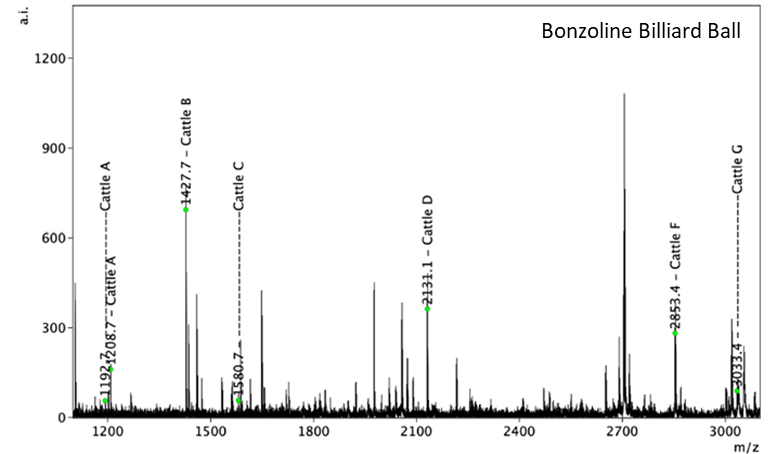
**

**Figure S10.** Peptide Mass Fingerprint MALDI spectra of the Bonzoline billiard ball. The cattle markers are indicated in the spectra.


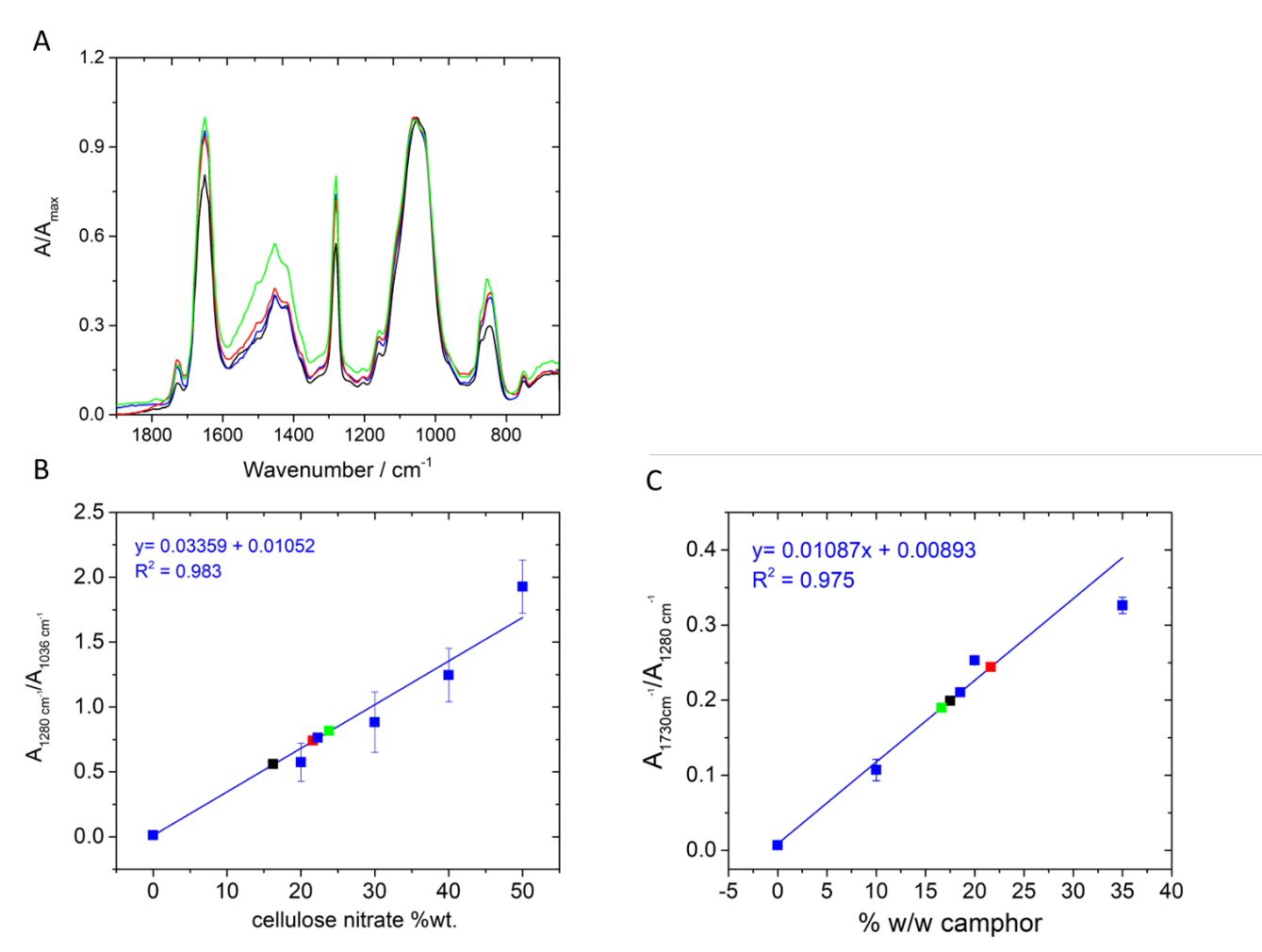


**Figure S11. Quantification of the bone-cellulose nitrate and camphor-cellulose nitrate formulation of a white Crystalate billiard ball.** A) Overlay of the infrared spectra of the four microsamples used for the quantification of Crystalate formulation, all acquired for the surface of the ball. The spectra are normalized to the maximum (νPO_4_^2-^ (~1036 cm^-1^) band. C) The cellulose nitrate concentration calculated for the four samples are showed (average 21%, standard deviation 2%). D) The camphor concentrations calculated for the three regions are showed (average 19%, standard deviation 1%).

**
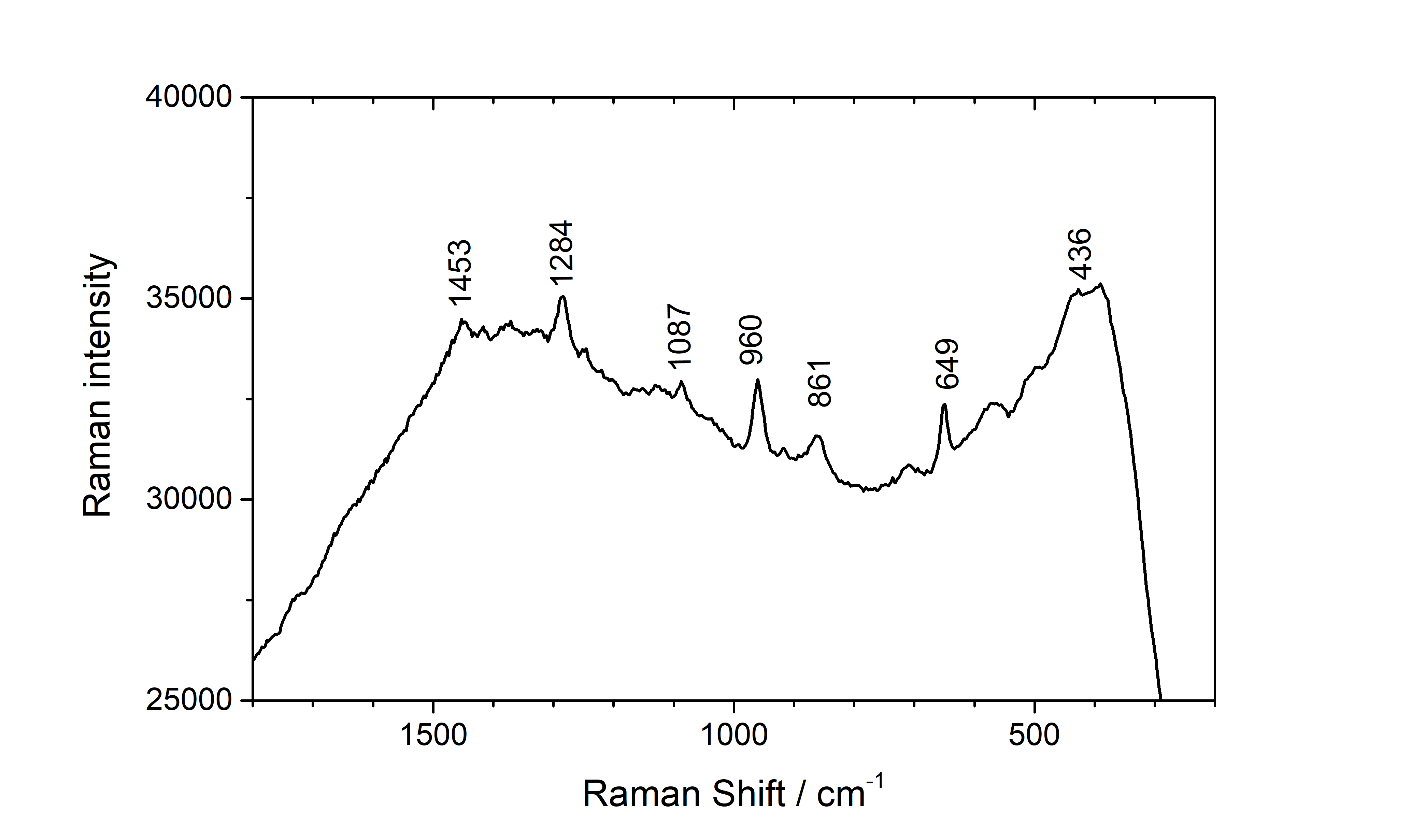
**

**Figure S12.** Handheld Raman Mira DS spectrum of the white Bonzoline billiard ball. (785nm, 7s acquisition time, 10 cycles). The bands of cellulose nitrate groups are observed at 861, 1284 and 1453 cm^-1^, of camphor at 649 cm^-1^, hydroxyapatite at 960 cm^-1^, calcite at 1087 cm^-1^ and zinc oxide at 436 cm^-1^.


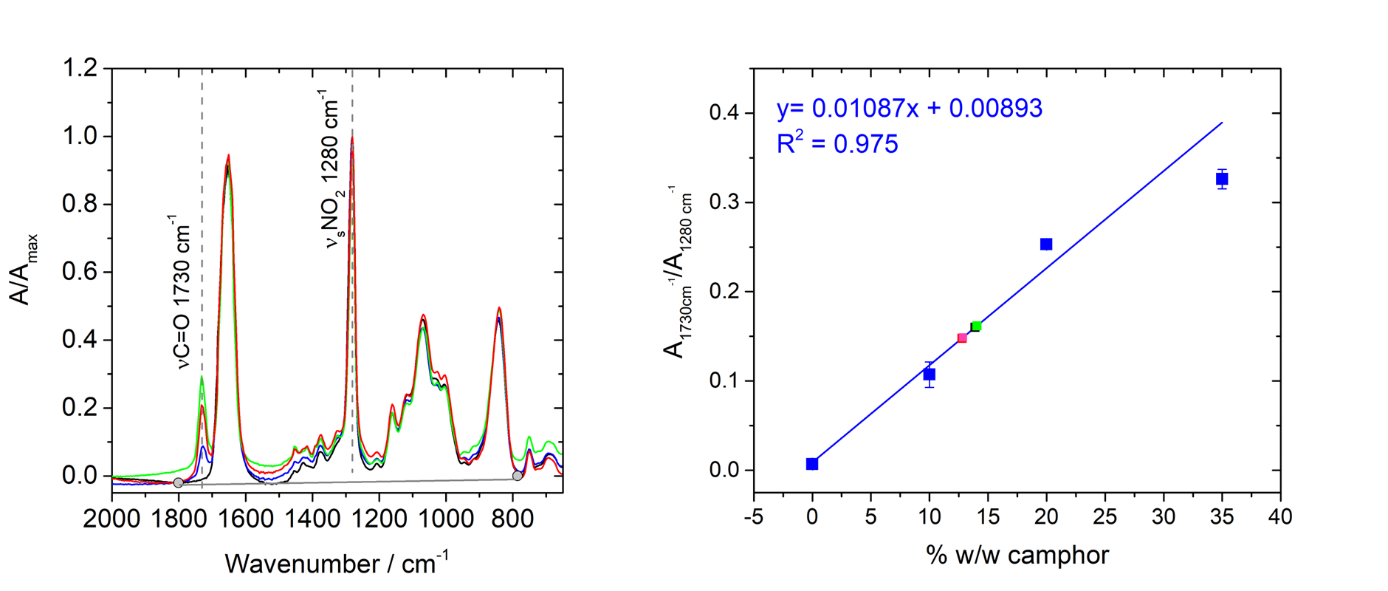


**Figure S13. Development of the camphor-cellulose nitrate calibration line and quantification of the plasticizer concentration in the 1868 billiard ball.** A) Overlay of the infrared spectra of the cellulose nitrate-camphor mixtures, normalized to the maximum (1280cm^-1^) (%wt. camphor/cellulose nitrate): 100% cellulose nitrate (black), 10/90 (blue), 20/80 (red), 35/65% (green). For the calibration curve, it was used the ratio between the carbonyl band of camphor, at 1730 cm^-1^, and the n_a_NO_2_ band of the cellulose nitrate, at 1280 cm^-1^. B) Calibration curve and respective equation and coefficient of determination (R^2^) for the linear fitting of the νC=O 1730 cm^-1^/ νNO_2_ 1280 cm^-1^ ratio calculated from µFTIR in function of the % w/w of camphor. The data shows the average ratio and the standard deviation calculated from the 3 infrared spectra collected for each reference. The colored points are the quantification of camphor in the 1868 billiard ball based on the analysis of 4 microsamples (average 13%, standard deviation 1%).

**
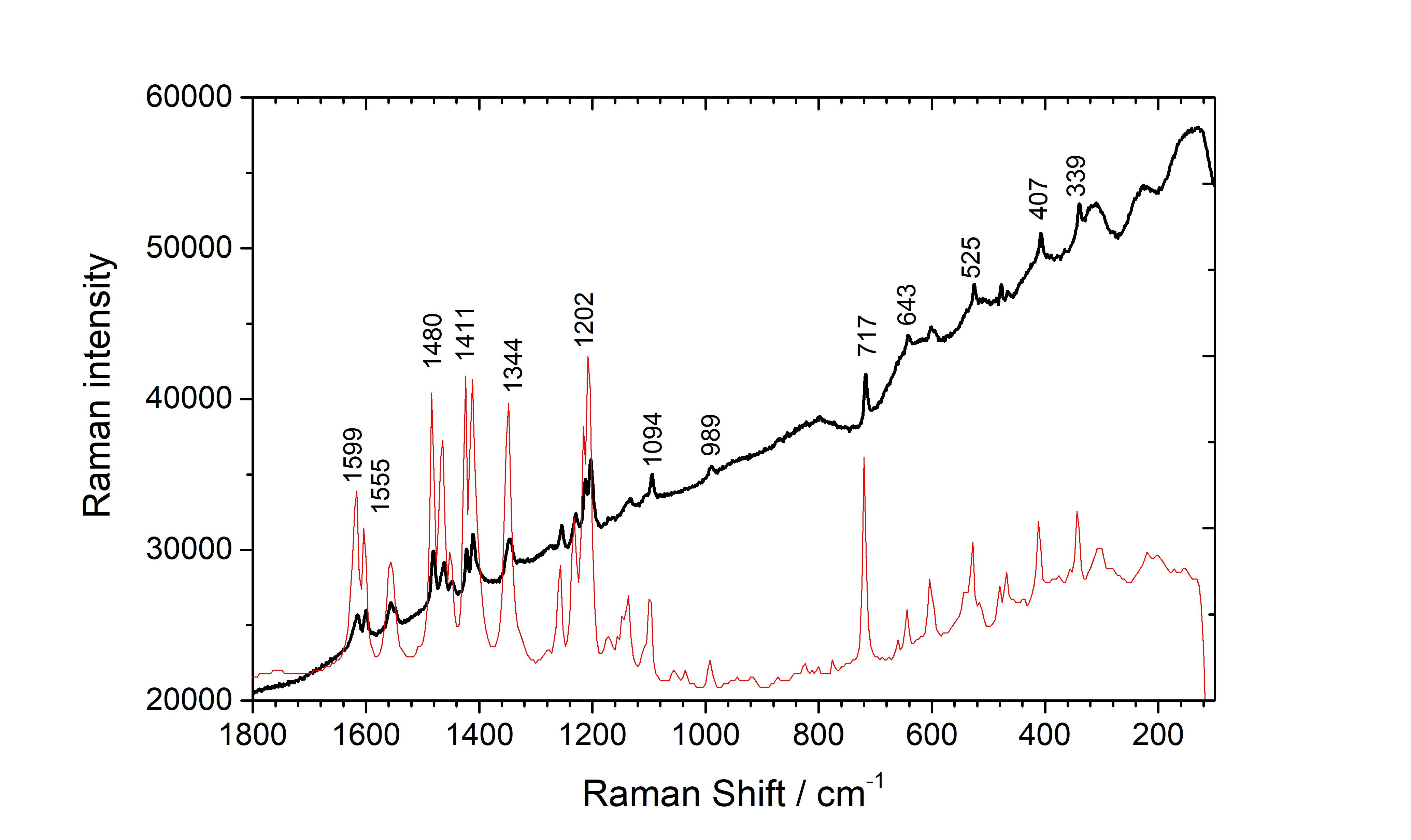
**

**Figure S14.** μRaman spectrum of the red Bonzoline ball at surface. Conditions: 633nm laser, 4.25 mW, 10s x 5 cycles acquisition time. Compared with a barium lithol red reference. Source: Herbst synthetic pigment database (in red, 633 nm)


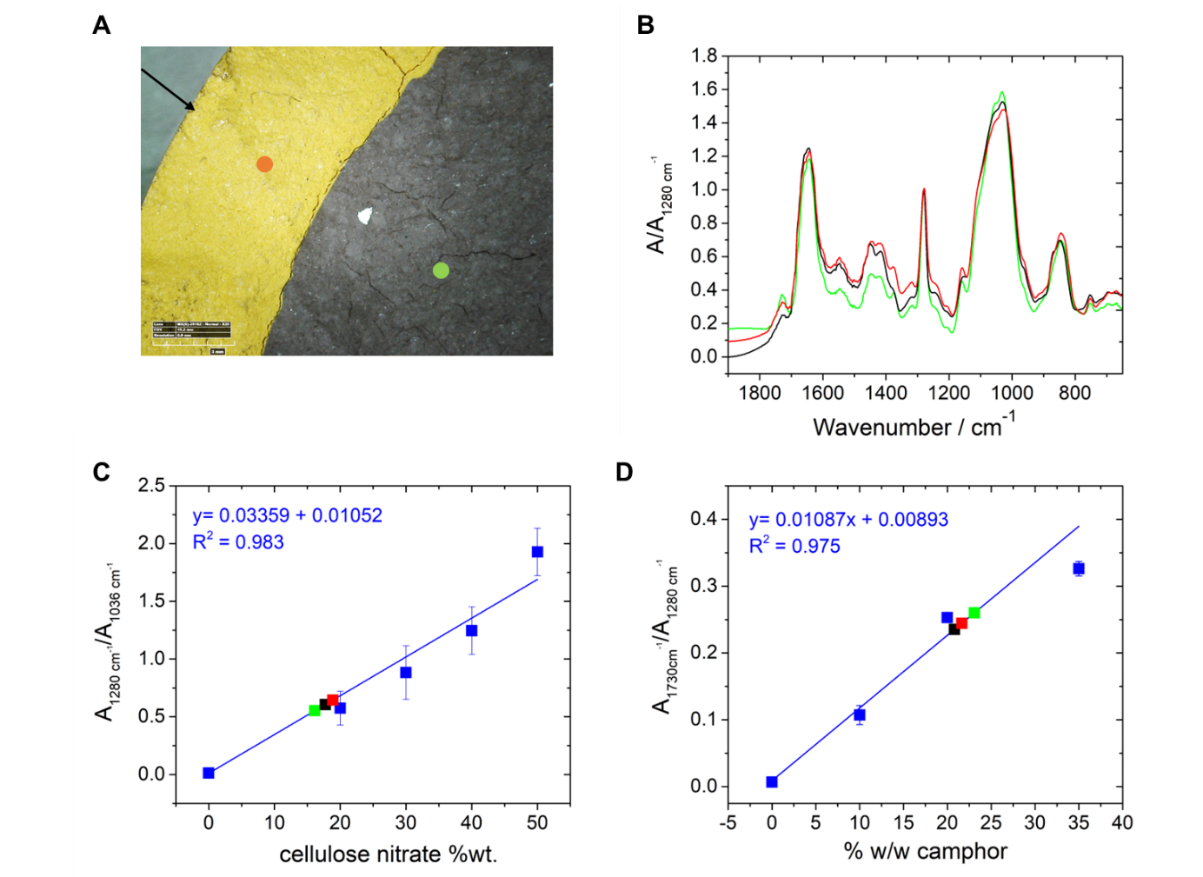


**Figure S15. Quantification of the bone-cellulose nitrate and camphor-cellulose nitrate formulation of the ivorylene billiard ball.** A) Areas of microsampling: surface (black), interior yellow (red) and brown core (green). B) Overlay of the infrared spectra of the three microsamples used for the quantification of ivorylene formulation. The spectra are normalized to the ν_s_NO_2_ (1280cm^-1^) band. C) The cellulose nitrate concentration calculated for the three regions are showed (average 17%, standard deviation 1%). D) The camphor concentrations calculated for the three regions are showed (average 22%, standard deviation 2%).


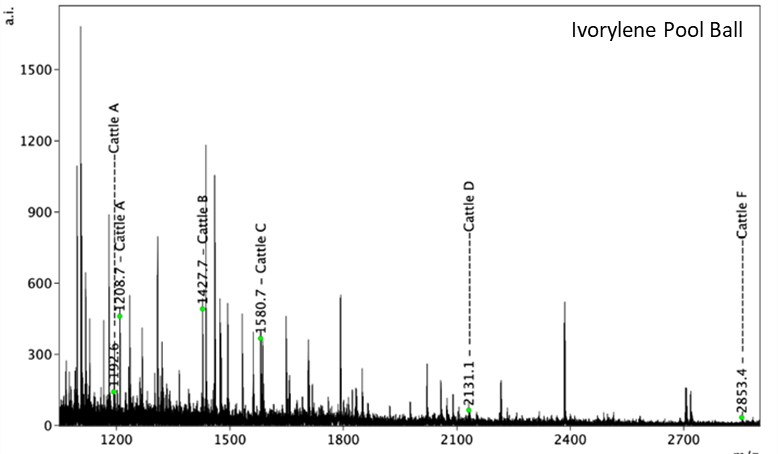


**Figure S16.** Peptide Mass Fingerprint MALDI spectra of the ivorylene pool ball. The cattle markers are indicated in the spectra

**
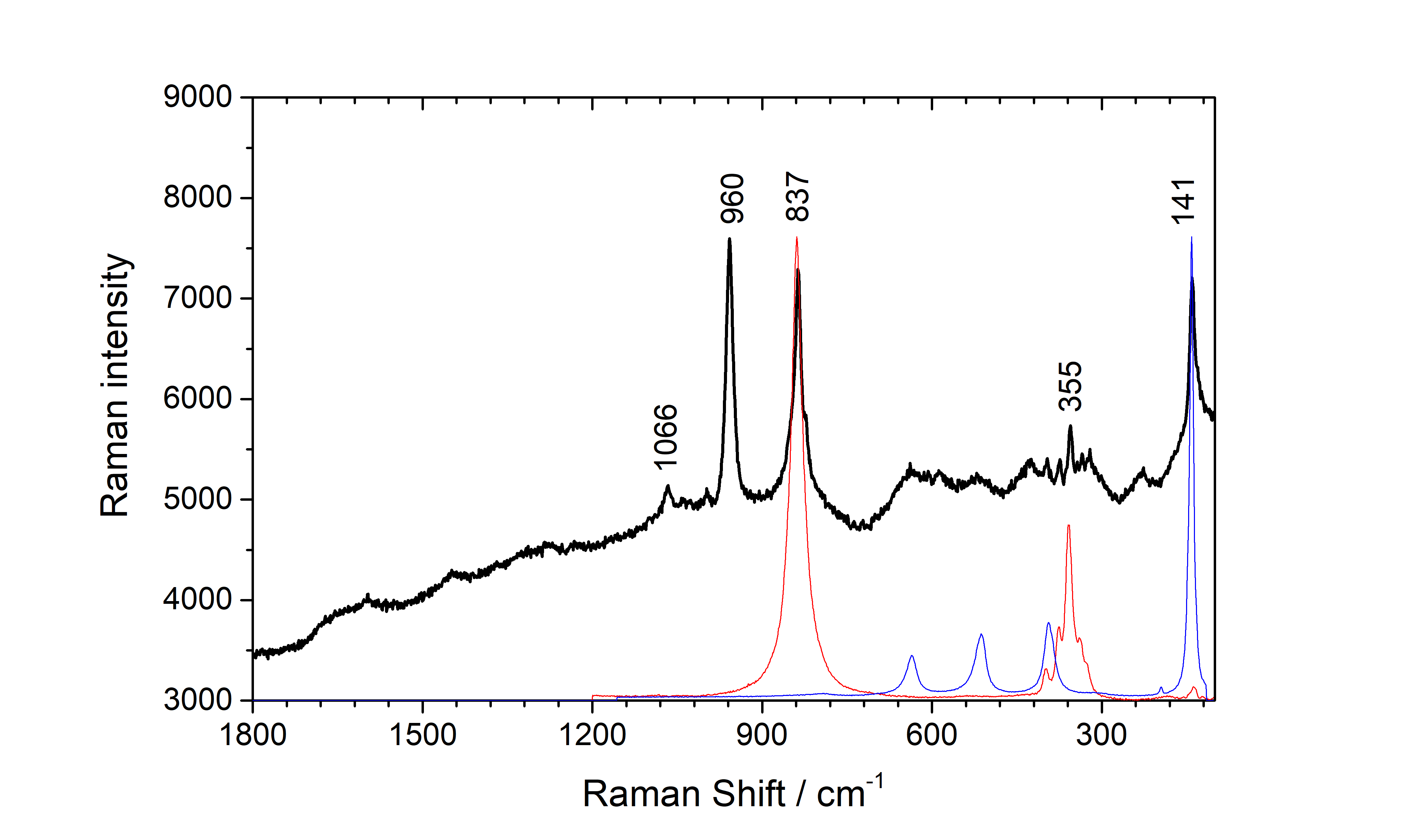
**

**Figure S17.** μRaman spectrum of the yellow ivorylene. Conditions: 633nm laser, 4.25 mW, 40s x 5 cycles acquisition time. Compared with a chrome yellow deep (PbCr_4_O.PbO) reference. Source: UCL London (in red, 633nm); and with and anatase (TiO_2_) reference. Source: Jobin-Yvon database (in blue, 633 nm). In more detail, chrome yellow was detected through its μRaman spectra by the observation of the chromate symmetric stretching ν_1_(CrO_4_^2-^) at 837 cm^-1^ and the symmetric bending ν_4_(CrO_4_^2-^) at 355 cm^-1^, (2). Anatase (TiO_2_) was identified by the detection of the characteristic strong and sharp band of the E_g_ mode at 140 cm^-1^ (3).


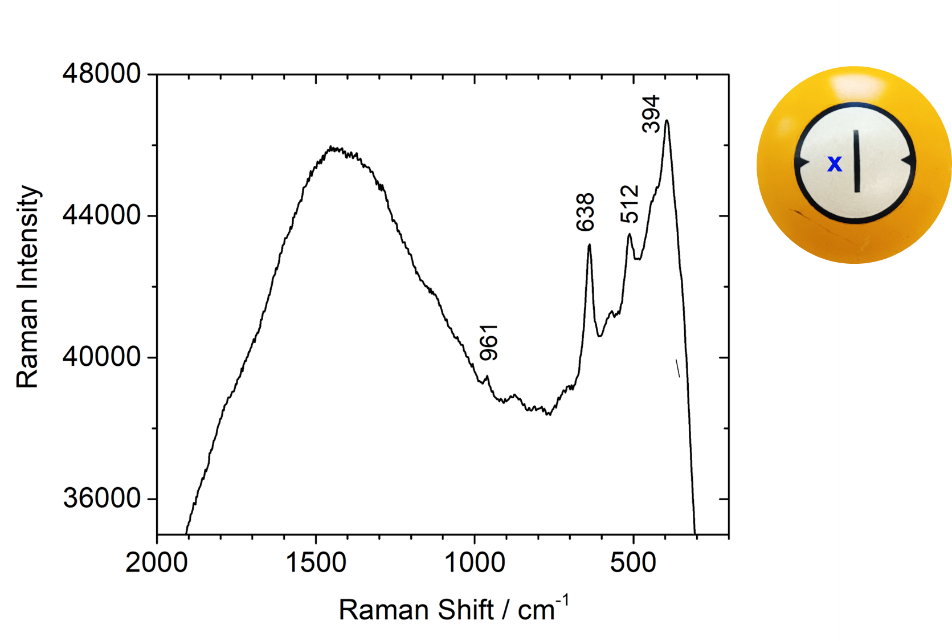


**Fig. S18.** Raman MIRA DS spectra obtained from the in-situ Raman MIRA DS analysis of the ivorylene pool ball white number region. The point of analysis is marked with a blue X on the pool ball image. Conditions: 0.5s acquisition time, 10 cycles. It was possible to detect anatase (TiO_2_), by the observation of bands at 394, 512 and 638 cm^-1^ (the strong band at 140 cm^-1^ is not observed due the equipment specificities). Raman MIRA DS was as an excellent tool to identify in-situ, bone, celluloid, chrome yellow and anatase in complex mixture.


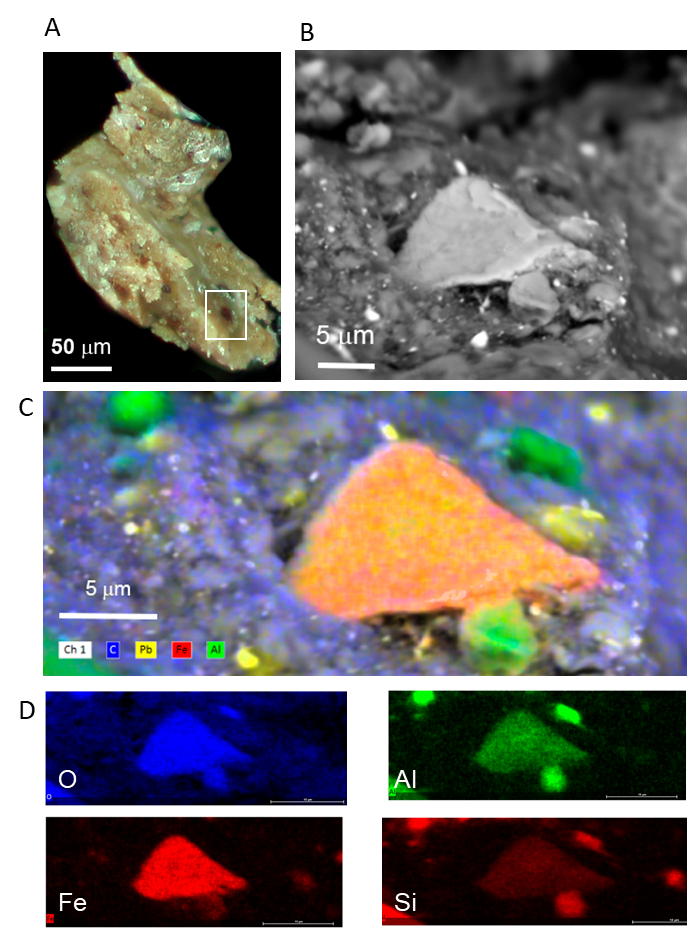


**Figure S19. SEM-EDS imaging of a dark particle observed in ivorylene’s brown core sample**. A) Microscope visible light image by SEM of the sample showing the location of the particle. B) Magnification showing the particle´s morphology. C) SEM-EDS electron beam induced X-Ray imaging showing the distribution of carbon, lead, iron and aluminum. D) Elemental component images, showing the emission of oxygen (O), aluminum (Al), iron (Fe) and silicon (Si). The dark particle showed significant concentrations of iron (Fe), aluminum (Al) and oxygen (O). Fe/Al oxides have dark toned colors, which correlated with the visual observation of the particle and overall core. These oxides are clay minerals, which indicates to the use of this material in the core of the ball. Around this particle were also found particles of lead (Pb), possibly from chrome yellow, and other unidentified aluminosilicates. In this ivorylene core region, EDS quantification of the concentration of magnesium (Mg) in the bone Ca-P rich phase (correlated to the overall distribution of oxygen) was found to be circa 0.3% mass% (elephant ivory has a higher concentration, around 4 %).


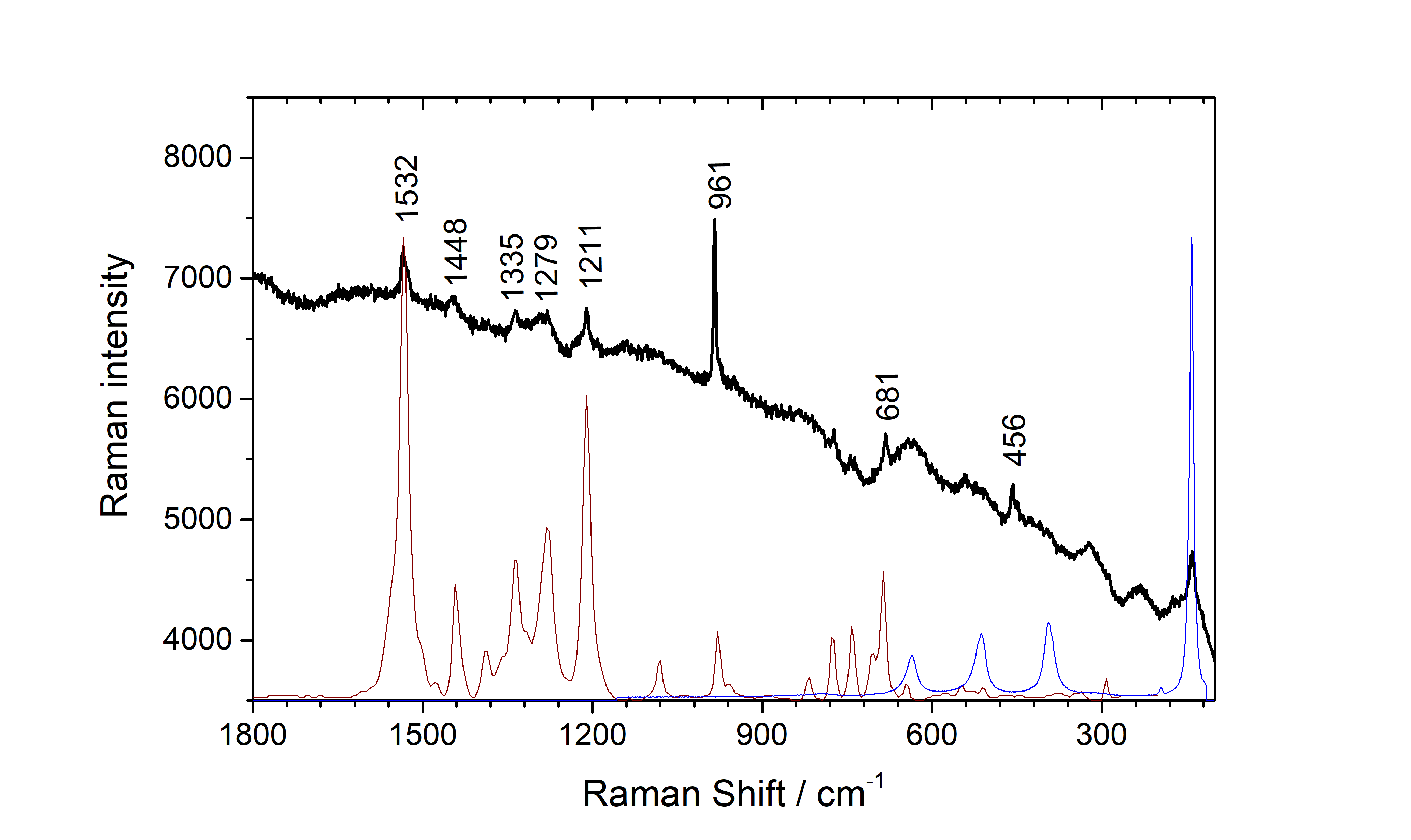


**Figure S20.** μRaman spectrum of the brown ivorylene core. Conditions: 633nm laser, 4.25 mW, 15s x 5 cycles acquisition time. Compared with copper phthalocyanine green G (PG7) reference. Source: UCL London (in brown, 633nm); and with and anatase (TiO_2_) reference. Source: Jobin-Yvon database (in blue, 633 nm).


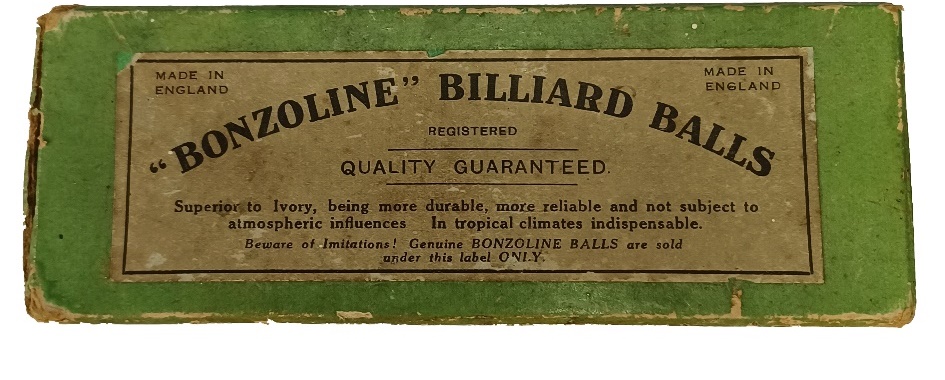

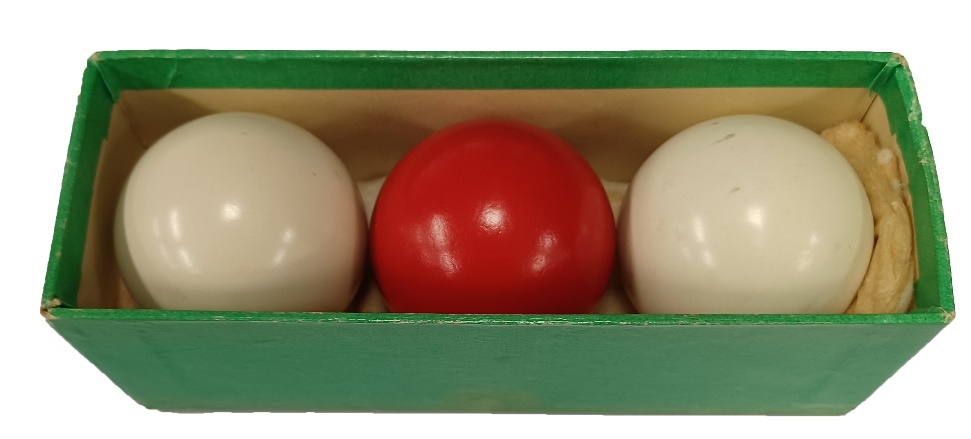


**Figure S21.** Photograph of the Bonzoline Billiard Balls set acquired in this work. In the top lid it is possible to read “Superior to ivory, being more durable, more reliable, and not subject to atmospheric influences. In tropical climates Indispensable.” The Bonzoline billiard balls started being manufactured in England in 1931 by the Composition Billliard Ball Supply Co., Stratford, London. Before that they were manufactured in the USA by the Albany Billiard Ball Company.


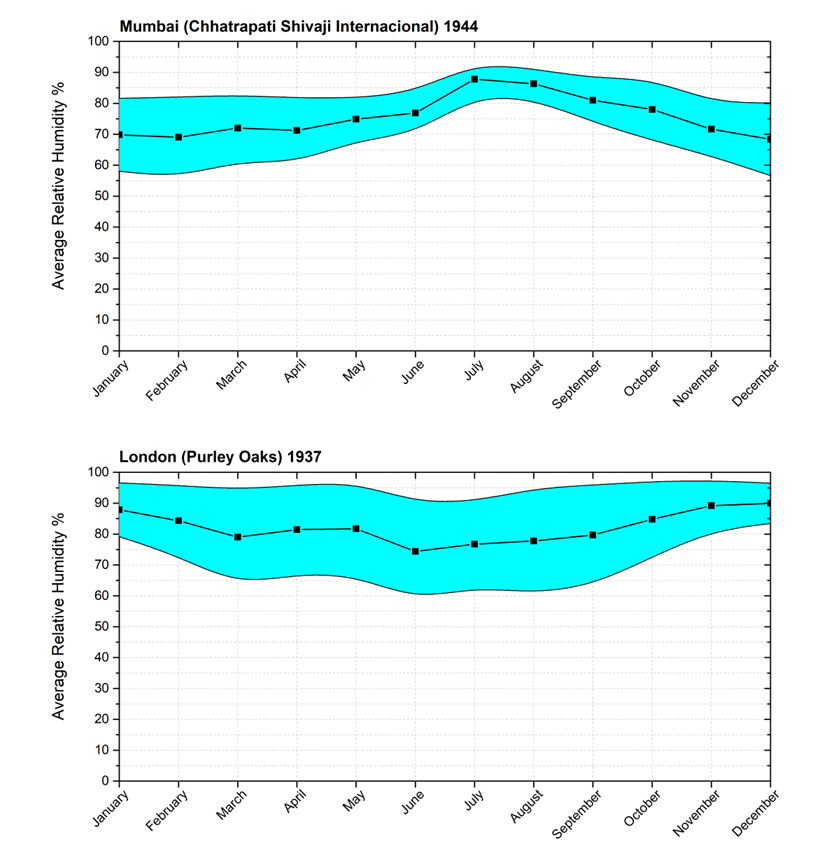


**Fig. S22.** **Yearly humidity variations (monthly averages and standard deviations) in Mumbai, India, and London, UK, in the years of 1944 and 1937, respectively.** The annual standard deviation (for Mumbai was 6.3% and for London 4.8%, which is demonstrative of the more prominent humidity fluctuations in the former. Data was acquired from Integrated Surface Dataset (Global) available at National Centers for Environmental Information. The stations (Chhatrapati Shivaji International and Purely Oaks) were selected based on their proximity to the city centers, and the years selected were the oldest available.


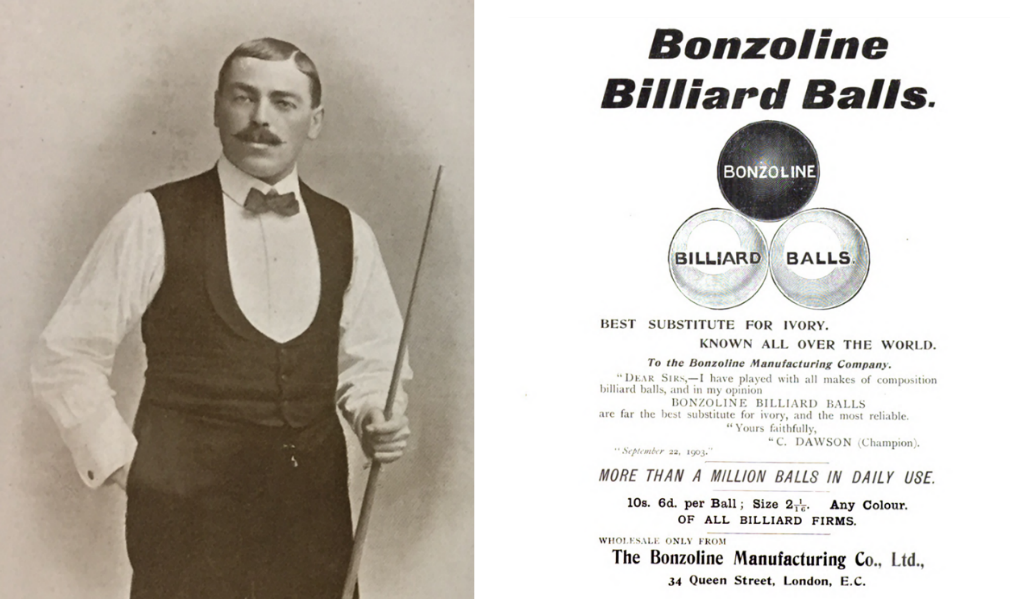


**Fig. S23.** **Charles Dawson, English billiard champion who defended ivory billiard balls in 1899 but later, in 1904, supported the sales of Bonzoline.**  Right, photo of Charles Dawson taken from the book he authored in 1904 “Practical Billiards”. Advertisement to the Bonzoline Billiard Balls in 1904, where it is possible to read the supportive statement of the Charles Dawson.


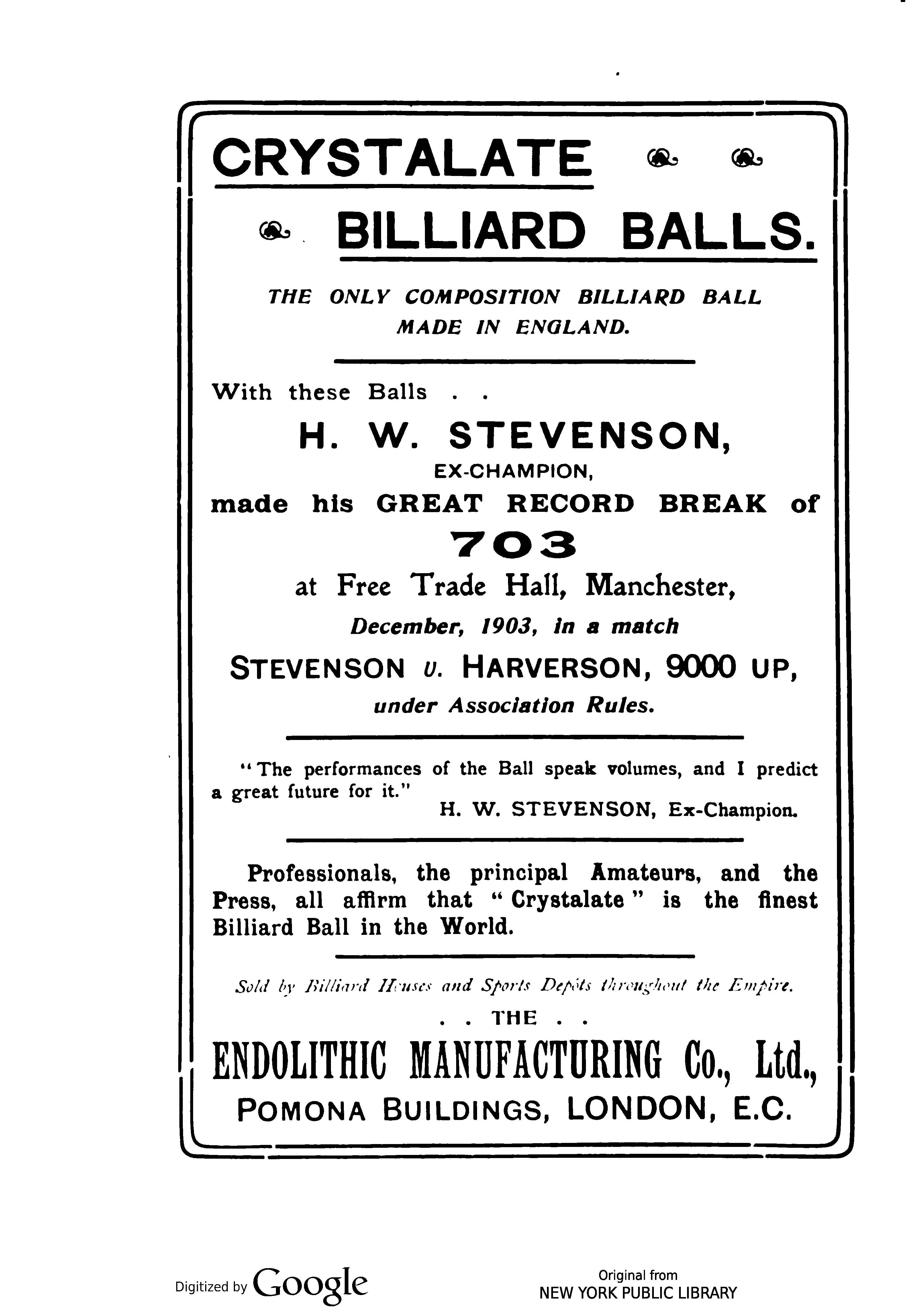

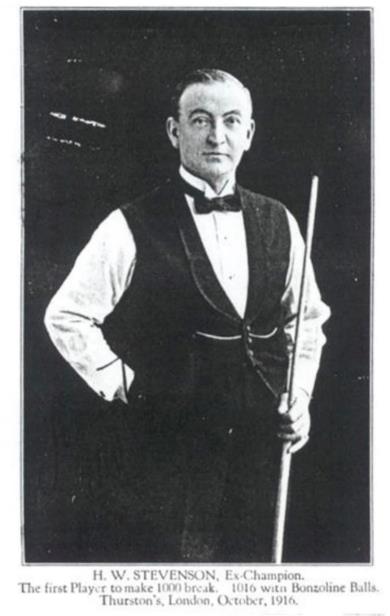


**Figure S24. Henry W. Stevenson, English billiards champion who played with Crystalate and Bonzoline balls.** Left, Henry W. Stevenson in 1916 when he made the first 1000 break with Bonzoline billiard balls. Right, 1904 advertisement to the Crystalate billiard ball with the referral to Henry W. Stevenson.


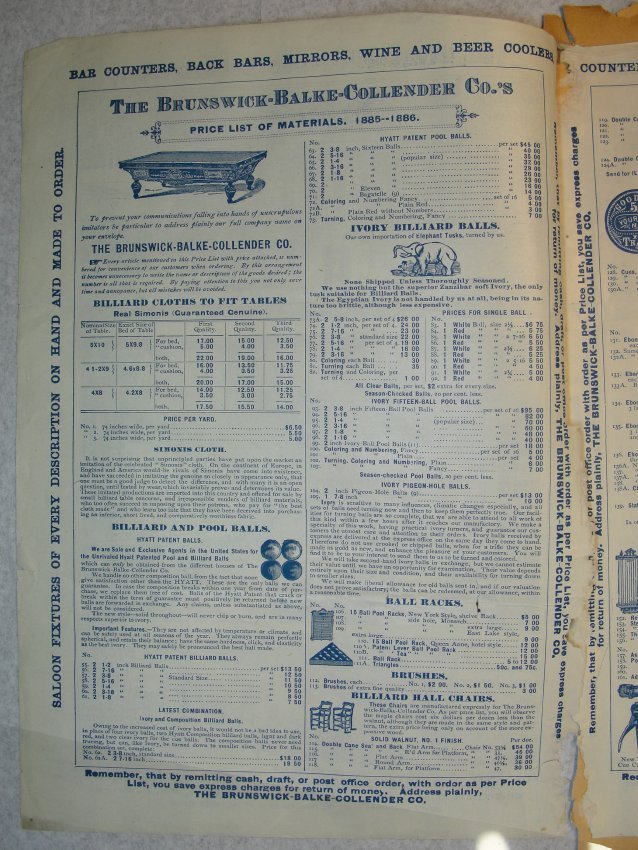


**Figure S25.** Page from the Brunswick – Balke – Collender Co. 1885 catalog with the price information for “Hyatt Patent Balls” and ivory balls (source: Brunswick digital library).


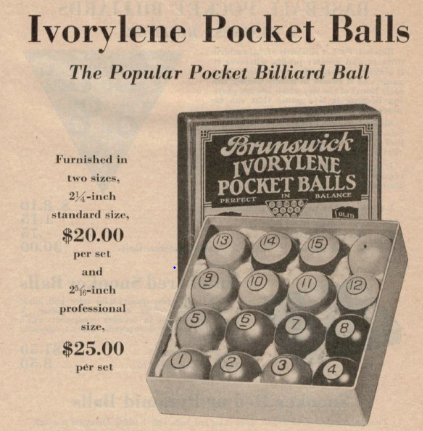


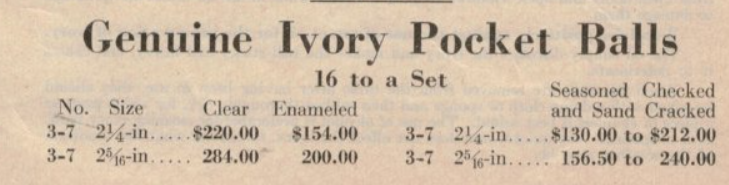


**Figure S26.** Clippings from the Brunswick – Balke – Collender Co. 1928 catalog with the prices of ivorylene and ivory pool sets (source: Brunswick digital library).


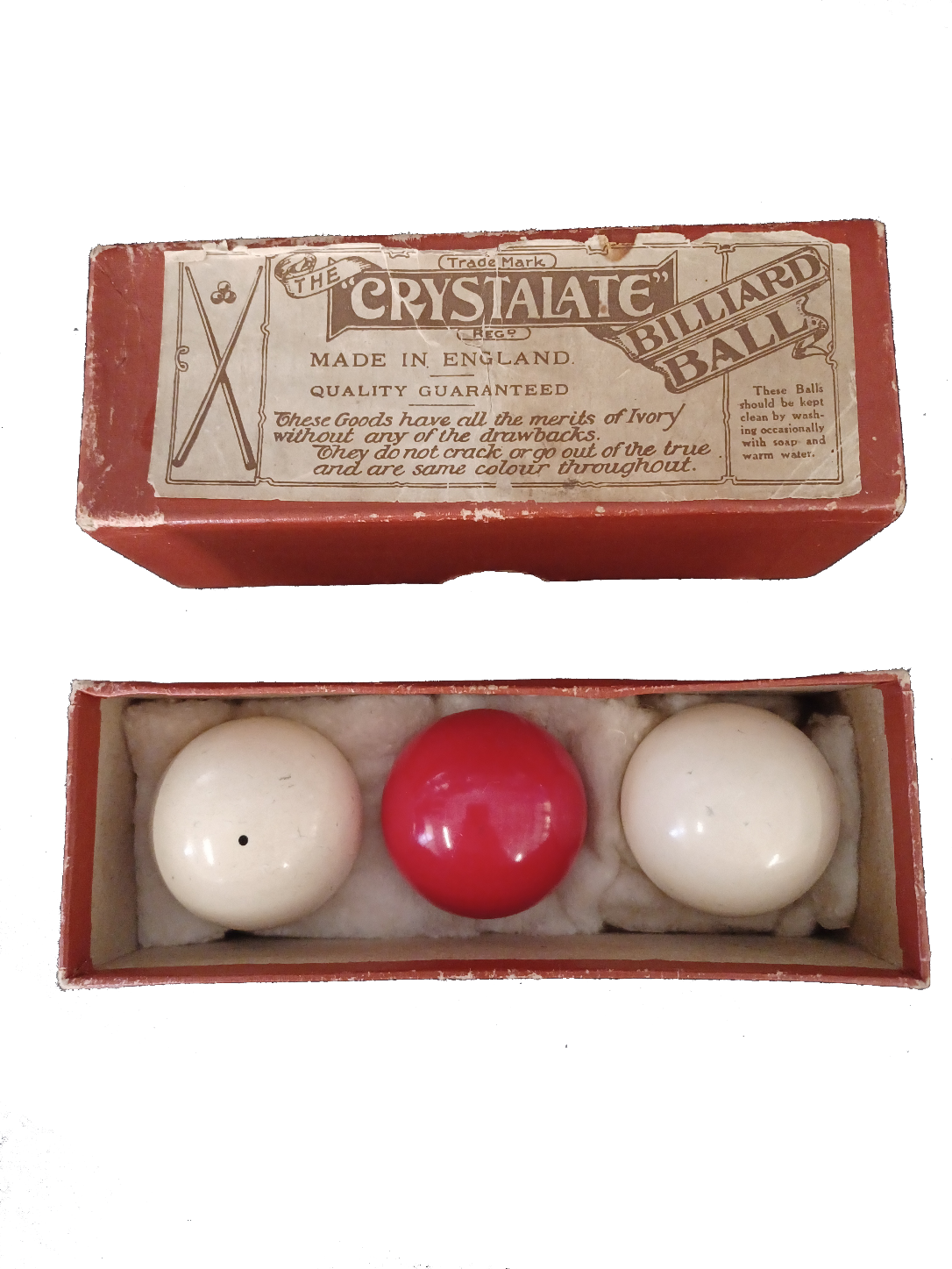


**Fig. S27.** Photograph of the Bonzoline Billiard Balls set acquired in this work. Weights from left to right: 73.559g, 73.348g, 73.089g.

**
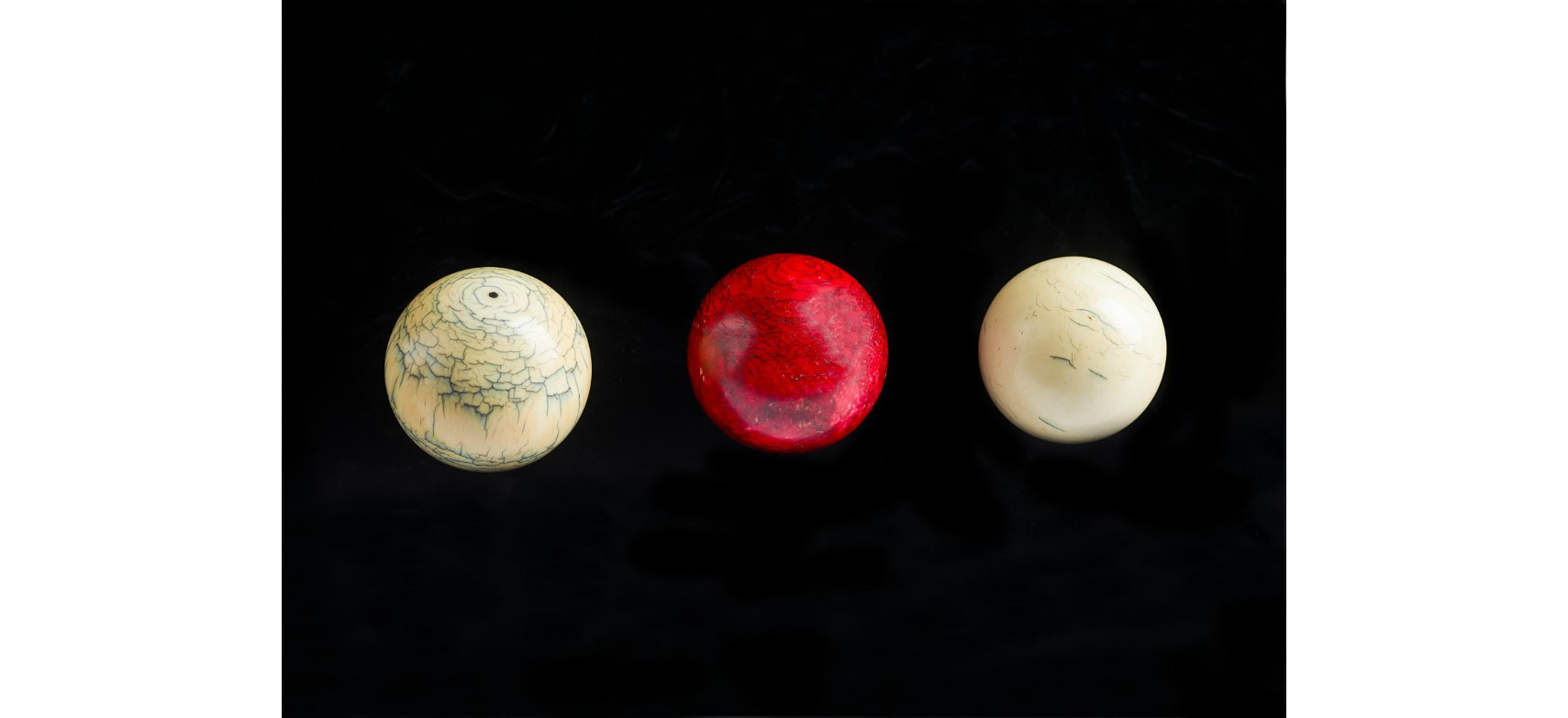
**

**Fig. S28.** Photograph of the NMAH’s ivory billiards balls, dated from 1875-1920 (ID Number CL.329507). The dimensions and weight of left and right billiard balls where measured: 57mm and 174.2g; 54mm and 143.8g, respectively.

Table S1. Transcriptions of the formulations for the manufacture of billiard balls and imitation ivory patented by John Wesley Hyatt and co-inventors from 1865 to 1915.

| **Title** | | **Formulation** | | **Patent** | **Company** |
| --- | --- | --- | --- | --- | --- |
| *Billiard-Balls* | | *I take* ***shellac*** *dissolved in alcohol and mix with it a sufficient quantity of* ***ivory-dust, or bone-dust****, or other ingredients, so as to form a paste which can be spread. For white balls I take bleached shellac and mix some* ***white lead or other white paint*** *with the ivory or bone-dust; and for colored balls I mix* ***vermillion or other colored paint*** *with the above-described composition. For colored balls the shellac may be dark* | | J.W. Hyatt, **1865,** USP 50359 | - |
| *Improvement in compositions for billiard balls and other articles* | | *First, I take* ***paper-pulp****, thoroughly saturated with water, so that it is in a semi-fluid condition. While in this condition I mix with it the flour of* ***gum-shellac, or any other similar fusible water-proof gum****, by thoroughly stirring them together. The proportions of the gum to the paper-pulp are about equal in quantity by weight in a dry state(…)The specific gravity of the composition may be adjusted by the addition of* ***white lead, or any suitable pigment****, to the gum (…)To color the composition, the pulp may be colored as desired before use, or* ***aniline red, or other coloring matter which will dissolve in alcohol*** *(…)* | | J.W. Hyatt, **1868,** USP 76765 | - |
| *Improved molding composition to imitate ivory and other compositions* | | ***Take any kind of fibrous vegetable, animal, or even mineral matter, such, for instance, as paper, leather-chips, or asbestus****, and reduce the same to a very fine state (…) I also use* ***gum-shellac, or any other solid, fusible, and adhesive gum or substance*** *(…)* *and reduce the same to a very fine powder. This cement I thoroughly intermix (…) both being in as dry a state The proportions of the cementing substance to the fibrous material are about equal in quantity by weight, although I do not confine myself to these proportions (…) If desirable, the specific gravity of the article or articles can be regulated by the use of* ***white lead or other suitable pigment.*** | | J.W. Hyatt, **1869,** USP 88633 | The Hyatt Mfg. Co., Albany, New York |
| *Improved method of coating billiard-balls* | | *I take a ball which is made of composition, or a substance of inferior appearance, and suspend it between two points, so that it can be rotated freely about its axis. While thus held the ball is* ***dipped into a solution of collodion****, and when removed therefrom it is rotated slowly, to cause the collodion to flow evenly over its surface, and when dry to form thereon a thin skin or coating.* | | J.W. Hyatt, **1869,** USP 88634 | The Hyatt Mfg. Co., Albany, New York |
| *Improved compound of ivory dust and other materials* | | *We form the collodion by taking say****, one pound of guncotton and*** *dissolving it in a mixture of equal parts of alcohol and ether, in sufficient quantity to produce a thick solution. With this solution we* *mix* ***three pounds of ivory-dust,*** *pulverized very fine and purified, forming the whole into a plastic mass.* | | J.W. Hyatt and D. Blake, **1869,** USP 89582 | - |
| *Improved method of making solid collodion* | | *We place soluble cotton, pyroxyline, or prepared cellulose into a strong cylinder or suitably-shape mold. With the pyroxyline may be mixed ivory-dust, bone-dust, asbestos, flake-white, or any other desirable substance, according to the nature of the product required (…) the proportion of the solvent the pyroxyline is as five to ten, seven to ten, or equal parts, by weight, according to the nature and proportions of the compound.* ***When pyroxyline is used alone, from one-half to three-fourths, by weight, of solvent will be sufficient; but when ivory-dust or another material is added, a somewhat greater proportion of solvent will be required****.* | | J.W. Hyatt and I.S. Hyatt, **1869,** USP 91341 | - |
| *Improvement in treating and molding pyroxyline* | | *(…) we mix therewith finely pulverized gum-camphor in about the proportions of* ***one part (by weight) of the camphor to two parts of the pyroxyline*** *when in a dry state. This proportions may somewhat be varied with good results.* | | J.W. Hyatt and I.S. Hyatt, **1870,** USP 105338 | - |
| *Improvement in processes of coating billiard-balls, knife-handles and other articles* | *I prepare the collodion by mixing sufficient alcohol and ether with guncotton to make a thick plastic mass, something like the consistency of dough, and then mold it upon the article to be coated therewith.* | | J.W. Hyatt, **1871,** USP 114945 | | - |
| *Improvement in factious ivory* | *We take, say,* ***one hundred parts by weight of ivory dust, one hundred parts of pyroxyline****,(by which term we mean soluble nitrocellulose)****, and fifty parts of powdered gum-camphor.*** | | J.W. Hyatt and I.S. Hyatt, **1874,** USP 156354 | | The Celluloid Mfg. Co., New York |
| *Improvement in silicious materials to imitate ivory and similar substances* | *To finely-pulverized* ***bone, horn, hoof, ivory, ivory-nut, or other similar substance containing gluten, albumen, and animal oils*** *(…)* ***added two (2) equal portions, by weight, of a solution of any of the alkaline silicates*** *- silicate of soda preferred – the silicate being of the consistency of syrup or molasses* | | J.W. Hyatt and C. M. Hyatt, **1878,** USP 201348 | | - |
| *Factitious material to imitate ivory, horn, etc.* | *The complete formula, therefore, will be,* ***of bone-dust, seventeen parts; of the solution of gum (four parts of bleach shellac, one part of borax and six parts of water), twenty-one parts; of boracic acid, one and one-half part.*** | | J.W. Hyatt, C.S. Lockwood, J.H. Stevens, **1880,** USP 236034 | | Bonsilate Company, Newark, New Jersey |
| *Manufacture of a factitious material to imitate ivory* | *(…) The solution will consist of* ***eight parts of shellac, thirty-two parts of ammoniacal water, and forty parts of zinc oxide.*** | | J.W. Hyatt, **1881,** USP 239794 | | - |
| *Plastic composition for the cores of billiard balls and for other purposes* | *In practicing the method last above referred to, I take a good article of* ***glue****, preferably in the form of powder, which is dissolved in a proper quantity of water by means of heat. After a solution has been formed, I add a percentage of* ***glycerine****, together with comminuted* ***bone*** *or its equivalent, and mix the elements in the most thorough manner possible, adding a pigment, earth or mineral matter, or coloring agent, if desired (…)* | | C.S. Lockwood, **1882**,USP259878 | | Bonsilate Company, (Limited), Albany, New York |
| *Process of and apparatus for molding plastic materials* | *The invention has relation to an improved process and apparatus to be used in the application of an exterior section or coating to articles having a core or interior portion made of material different from the exterior part. (no formulation is given)* | | J.W. Hyatt, C.S. Lockwood, **1882,** USP 259984 | | Bonsilate Company, (Limited), Albany, New York |
| *Plastic material* | *In practicing my invention, I take, say,* ***eight pounds of bone, ivory, horn, fish scales, quills, or other materials from the same nature*** *(…)* ***two ounces of phosphate of ammonia*** *(…)* ***two pounds of shellac*** *(…) and mix the elements thoroughly together.* | | C.S. Lockwood, **1883**,USP283793 | | Bonsilate Company, (Limited), Albany, New York |
| *Plastic material* | *The ingredients having been prepared, I take about* ***eight pounds of pulverized bone*** *and add to it about* ***two ounces of phosphate of ammonia.*** | | C.S. Lockwood, **1884**, USP 283793 | | Bonsilate Company, (Limited), Albany, New York |
| *Game-Ball with composition coating* | *This invention relates to the manufacture of balls having a composition coating which is molded while in a plastic condition upon a hard core and allowed to harden and season there on.* ***Celluloid, casein compounds, hard rubber, and other compositions*** *are used for such purposes.* | | J.W. Hyatt and C. H. Hyatt, **1915**, 1156144 | | - |

**SI References**

1. I. Kontopoulos, S. Presslee, K. Penkman, M. J. Collins, Preparation of bone powder for FTIR-ATR analysis: The particle size effect. *Vib Spectrosc* **99**, 167–177 (2018).

2. L. Monico, K. Janssens, E. Hendriks, B. G. Brunetti, C. Miliani, Raman study of different crystalline forms of PbCrO4 and PbCr1-xSxO4 solid solutions for the noninvasive identification of chrome yellows in paintings: a focus on works by Vincent van Gogh. *Journal of Raman Spectroscopy* **45**, 1034–1045 (2014).

3. T. Ohsaka, F. Izumi, Y. Fujiki, Raman spectrum of anatase, TiO2. *Journal of Raman Spectroscopy* **7**, 321–324 (1978).
